# TET1 regulates hypoxia-induced epithelial-mesenchymal transition by acting as a co-activator

**DOI:** 10.1186/s13059-014-0513-0

**Published:** 2014-12-03

**Authors:** Ya-Ping Tsai, Hsiao-Fan Chen, Sung-Yuan Chen, Wei-Chung Cheng, Hsei-Wei Wang, Zih-Jie Shen, Chunxiao Song, Shu-Chun Teng, Chuan He, Kou-Juey Wu

**Affiliations:** Institute of Biochemistry & Molecular Biology, National Yang-Ming University, No. 112, Sec. 2, Li-Nong St., Taipei, 112 Taiwan; Research Center for Tumor Medical Science, Grad. Inst. of Cancer Biology, China Medical University, No. 91, Hseuh-Shih Rd., Taichung, 404 Taiwan; Institute of Microbiology & Immunology, National Yang-Ming University, Taipei, 112 Taiwan; Department of Microbiology, College of Medicine, National Taiwan University, Taipei, 100 Taiwan; Department of Chemistry and Institute for Biophysical Dynamics, University of Chicago, Chicago, IL 60637 USA; Howard Hughes Medical Institute, University of Chicago, Chicago, IL 60637 USA

## Abstract

**Background:**

Hypoxia induces the epithelial-mesenchymal transition, EMT, to promote cancer metastasis. In addition to transcriptional regulation mediated by hypoxia-inducible factors, HIFs, other epigenetic mechanisms of gene regulation, such as histone modifications and DNA methylation, are utilized under hypoxia. However, whether DNA demethylation mediated by TET1, a DNA dioxygenase converting 5-methylcytosine, 5mC, into 5-hydroxymethylcytosine, 5hmC, plays a role in hypoxia-induced EMT is largely unknown.

**Results:**

We show that TET1 regulates hypoxia-responsive gene expression. Hypoxia/HIF-2α regulates the expression of TET1. Knockdown of TET1 mitigates hypoxia-induced EMT. RNA sequencing and 5hmC sequencing identified the set of TET1-regulated genes. Cholesterol metabolic process genes are among the genes that showed high prevalence and statistical significance. We characterize one of the genes, INSIG1 (insulin induced gene 1), to confirm its expression and the 5hmC levels in its promoter. Knockdown of INSIG1 also mitigates hypoxia-induced EMT. Finally, TET1 is shown to be a transcriptional co-activator that interacts with HIF-1α and HIF-2α to enhance their transactivation activity independent of its enzymatic activity. TET1 acts as a co-activator to further enhance the expression of INSIG1 together with HIF-2α. We define the domain in HIF-1α that interacts with TET1 and map the domain in TET1 that confers transactivation to a 200 amino acid region that contains a CXXC domain. The TET1 catalytically inactive mutant is capable of rescuing hypoxia-induced EMT in TET1 knockdown cells.

**Conclusions:**

These findings demonstrate that TET1 serves as a transcription co-activator to regulate hypoxia-responsive gene expression and EMT, in addition to its role in demethylating 5mC.

**Electronic supplementary material:**

The online version of this article (doi:10.1186/s13059-014-0513-0) contains supplementary material, which is available to authorized users.

## Background

Cells develop various mechanisms to cope with hypoxia and survive [1,2]. Hypoxia induces the epithelial-mesenchymal transition (EMT) to promote cancer metastasis [3-6]. In addition to transcriptional regulation mediated by hypoxia-inducible factors (HIFs), other epigenetic mechanisms of gene regulation, such as histone modifications and DNA methylation, are utilized under hypoxia [7-9]. Certain chromatin changes have been observed during EMT. For example in Snail-induced EMT, loss of H3K4Me3, H3K4Ac, and H3K27Ac, and gain of H3K27Me3 were observed for genes repressed, whereas gain of H3K4Me3, H3K4Me1, and loss of H3K27Me3 were observed for genes activated [10]. Other specific chromatin changes have also been observed during hypoxia or TGF-β-induced EMT [11,12].

DNA demethylation is recently shown to be an important epigenetic mechanism that regulates gene expression due to the discovery of TET (ten-eleven translocation) enzymes that demethylate DNA [13,14]. TET enzymes have been shown to convert 5-methylcytosine (5mC) to 5-hydroxymethylcytosine (5hmC) to regulate gene expression [13-15]. Tet proteins can also convert 5-methylcytosine to 5-formylcytosine and 5-carboxycytosine [16,17]. Tet1 and Tet2 are regulated by Oct4 during somatic cell reprogramming into induced pluripotent stem cells [18]. However, other mechanisms regulating expression of Tet1 genes remain largely unknown.

In this report, we explored the mechanism of TET1 regulation by hypoxia. The role TET1 in regulating the process of EMT induced by hypoxia was investigated. The set of genes that was regulated by TET1 under hypoxia and their role in hypoxia-induced EMT were delineated. Finally, we showed the extra role of TET1 in serving as a transcription co-activator. These results provide a fresh insight into regulation of hypoxia-responsive gene expression by TET1 and further expand the role of TET1.

## Results and discussion

### Hypoxia/HIF-2α activates TET1 expression and knockdown of TET1 mitigates hypoxia-induced epithelial-mesenchymal transition

**Figure 1:**
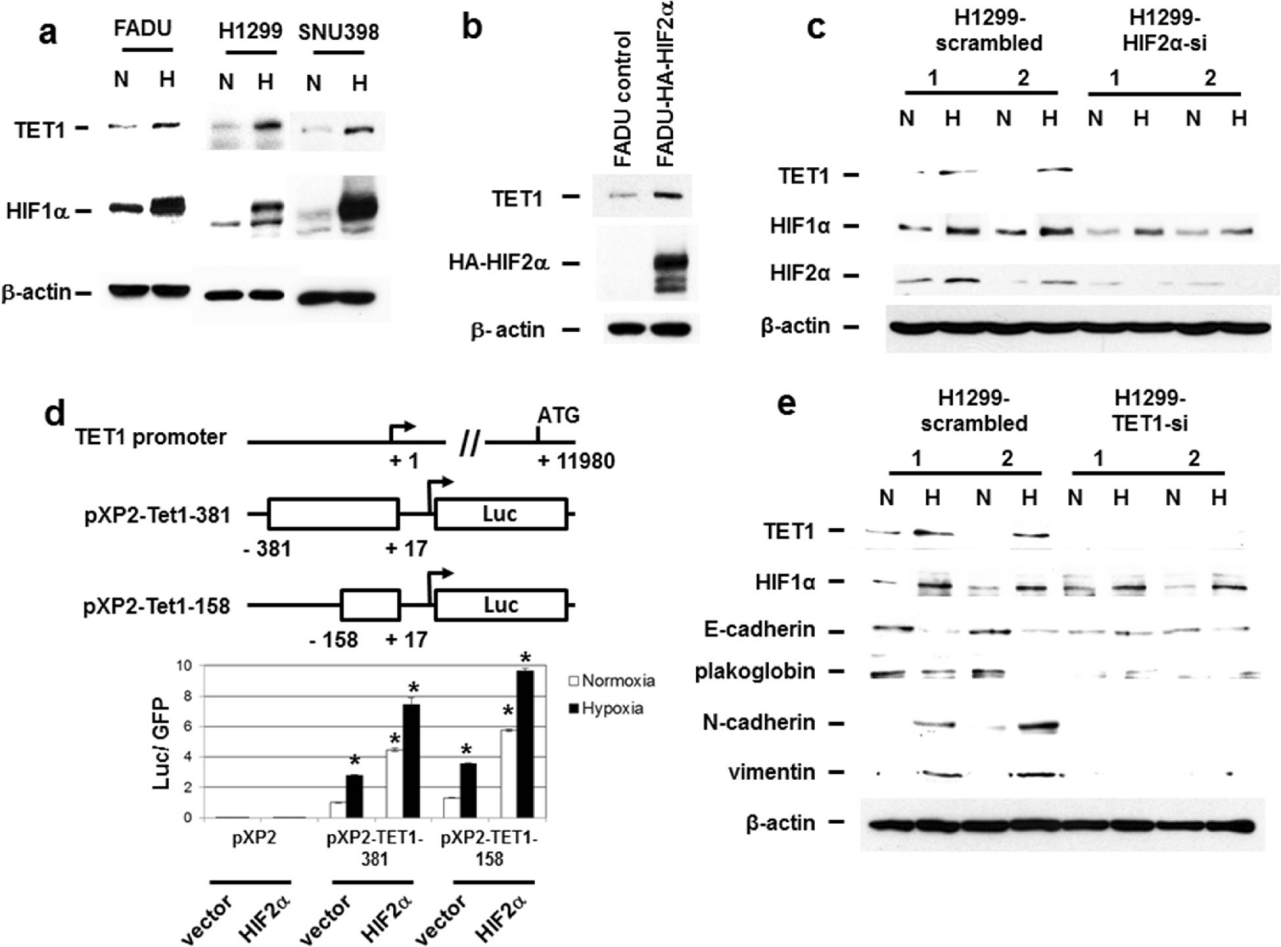
**Activation of TET1 by hypoxia/HIF-2α and knockdown of TET1 mitigated hypoxia-induced epithelial-mesenchymal transition. (a)** Western blot analysis showed the activation of TET1 by hypoxia. N: normoxia; H: hypoxia. **(b)** Overexpression of HIF-2α activated TET1 expression using western blot analysis. **(c)** Knockdown of HIF-2α abolished the activation of TET1 by hypoxia. **(d) **Reporter gene assays showed the activation of *TET1* proximal promoter by HIF-2α. The asterisk (*) indicates statistical significance (*P* <0.05) between experimental and control transfections. pXP2-Tet1-381 or pXP2-Tet1-158 vector alone was used as the control transfection. Error bars indicate standard deviations (s.d.) of triplicate luciferase activity. **(e)** Knockdown of TET1 mitigated the process of hypoxia-induced EMT.

TET enzymes have been shown to convert 5mC to 5hmC to regulate gene expression [13-15]. In spite of the various epigenetic mechanisms demonstrated to regulate hypoxia-responsive gene expression [8,9], the role of TET enzymes and DNA demethylation in regulating hypoxia-responsive genes remain largely unknown. We tested whether TET1 could be regulated by hypoxia to mediate hypoxia-regulated gene expression. Exposure of various cell lines to hypoxia showed the activation of *TET1* mRNA expression (Additional file 1a). Western blot analysis confirmed the upregulation of TET1 protein levels by hypoxia (Figure 1a). Overexpression of HIF-2α, but not HIF-1α, activated the expression of TET1 (Figure 1b and data not shown). Knockdown of HIF-2α abolished the activation of TET1 under hypoxia in two different cell lines (Figure 1c and Additional file 1b and c), indicating that HIF-2α was the major regulator of TET1 expression under hypoxia. We further identified the region in the proximal promoter of *TET1* gene that responded to hypoxia and HIF-2α. Reporter gene assay showed that the promoter region (-381 to +17 bp upstream of the transcription start site, TSS) of the *TET1* gene was activated by hypoxia/HIF-2α (Figure 1d and Additional file 2). The hypoxia response region was further narrowed down (-158 to +17 bp upstream of the TSS) (Figure 1d). The construct containing the promoter region of -91 to +17 bp upstream of the TSS did not respond to hypoxia/HIF-2α stimulation (data not shown), further narrowing down the hypoxia/HIF-2α responsive region to -158 to -91 bp upstream of the TSS of *TET1* gene. Chromatin immunoprecipitation experiments showed that HIF-2α directly bound to the proximal promoter region (Additional file 1d). Positive control also showed that HIF-2α bound to the promoter region of *WDR5* gene (Additional file 1d) [11]. All these results indicate the direct regulation of *TET1* gene expression by HIF-2α.

Since EMT is induced by hypoxia to promote tumor metastasis [3-6], we tested whether TET1 plays a significant role in hypoxia-induced EMT. TET1 knockdown was performed in a H1299 lung cancer cell line. Exposure of H1299 cells to hypoxia induced EMT as characterized by repression of E-cadherin/plakoglobin and upregulation of vimentin/N-cadherin expression [5,6] (Figure 1e). Knockdown of TET1 abolished the upregulation of mesenchymal gene expression including vimentin and N-cadherin (Figure 1e). Immunofluorescence assays showed the no change of E-cadherin expression and abolishment of vimentin upregulation in H1299 cells under TET1 knockdown (Additional file 3a). TET1 knockdown also significantly decreased the *in vitro* migration and invasion activity induced by hypoxia in H1299 cells (Additional file 3b). The same results were obtained using the head and neck cancer FADU cell line (Additional file 4). These results indicate that TET1 plays a crucial role in hypoxia-induced EMT and induction of *in vitro* migration/invasion activity.

### Cholesterol metabolic process is regulated by TET1 and knockdown of INSIG1 mitigates hypoxia-induced epithelial-mesenchymal transition

**Figure 2:**
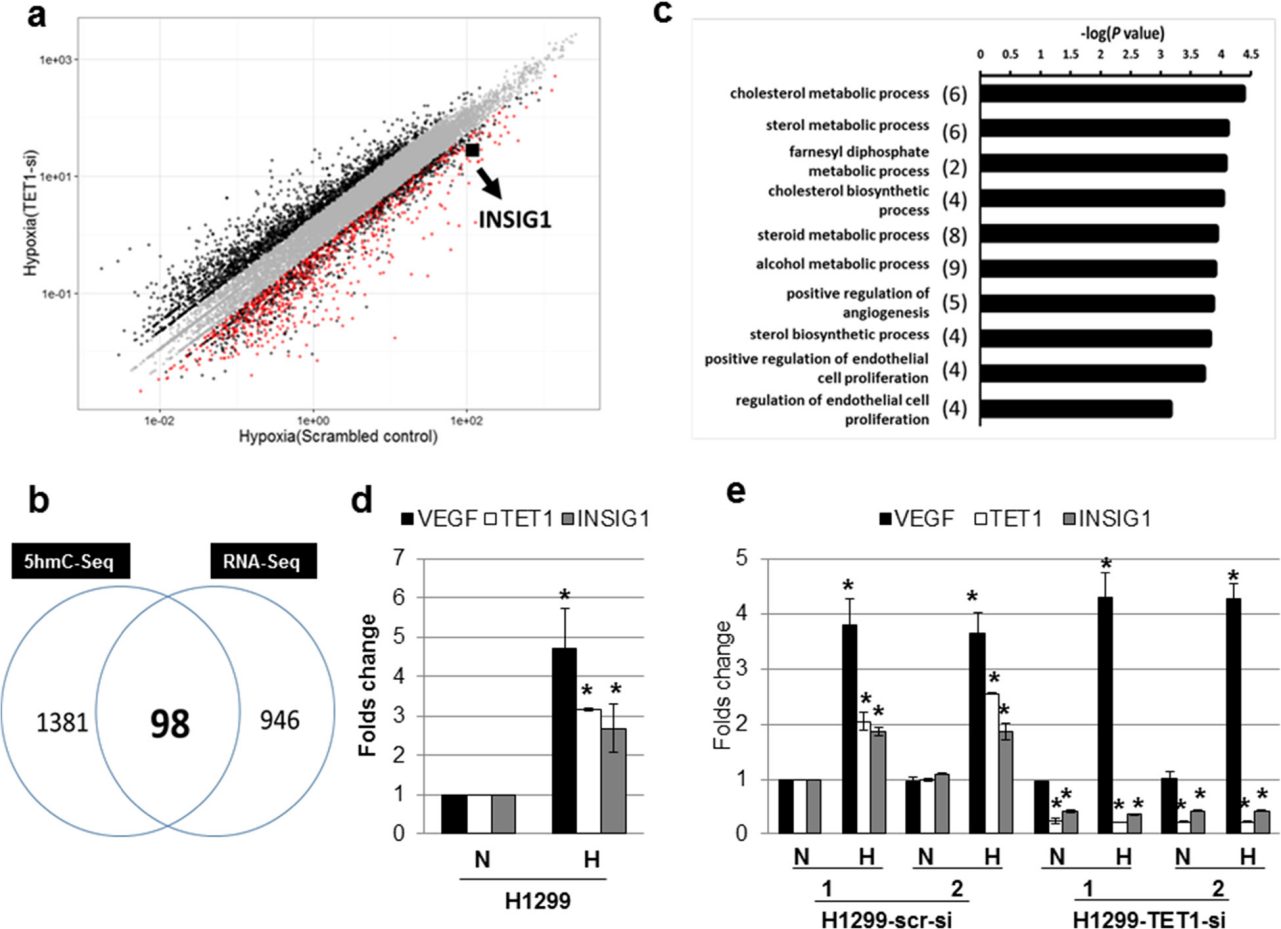
**Cholesterol metabolic process was the major pathway regulated by hypoxia-induced TET1 and activation of** ***INSIG1*** **expression by hypoxia, which was abolished by TET1 knockdown using real-time PCR analysis. (a)** Scatter plot comparing transcriptome profile of hypoxia sample with scrambled knockdown vs. hypoxia sample with TET1 knockdown in FADU cell lines. Gene expression was measured by FPKM. The genes with the fold change >2 or <0.5 were denoted as black dots. The 1,044 genes regulated by TET1 under hypoxia were highlighted by red dots. The square dot indicating the *INSIG1* gene was pointed out. **(b)** Venn diagram showed the overlapping group of genes (98) that had both differential RNA expression and differential 5hmC-enriched promoter regions. **(c)** Gene Ontology analysis of the genes with differential 5hmC-enriched regions and differential RNA expression. The top 10 enriched biological processes based on their *P* values were shown. Cholesterol metabolic process ranked the most significant group of genes regulated according to RNA expression and 5hmC peak levels. **(d)** Activation of *INSIG1* expression by hypoxia in H1299 cells using real-time PCR analysis. N: normoxia; H: hypoxia. **(e)** Knockdown of TET1 abolished the activation of *INSIG1* expression induced by hypoxia in H1299 cells using real-time PCR analysis. The asterisk (*) indicates statistical significance (*P* <0.05) between experimental and control clones. The H1299 vector or H1299 scrambled control clone under normoxia was chosen as the control condition in (d) and (e). Error bars indicate standard deviations (s.d.) of duplicate mRNA levels by real-time PCR analysis **(d,e)**.

Since TET1 demethylates 5mC to regulate gene expression [13,14], we wanted to determine the genes whose expression was regulated by TET1 under hypoxia. RNA sequencing experiments using FADU control vs. FADU-TET1 knockdown cells under normoxia or hypoxia showed that a total of 1,044 genes were regulated by TET1 under hypoxia since their expression decreased under TET1 knockdown (Figure 2a and b). Transcriptome profiles were shown in Figure 2a. Gene Ontology (GO) analysis of these genes showed that they belonged to various biological processes (Additional file 5a). To further narrow down the genes that were regulated by TET1 and also showed changes in the levels of 5hmC in their promoter regions, 5hmC sequencing was performed on these FADU clones under normoxia or hypoxia (control vs. TET1 knockdown) [19]. Analysis of 5hmC sequencing results showed the distribution of 5hmC peaks (Additional file 5b). There were no genome-wide changes of 5hmC labeling with regards to their genomic locations under normoxia or hypoxia (Additional file 6a), indicating that 5hmC regulated by TET1 only influenced the expression of certain specific genes. To search for genes that were regulated by TET1 from RNA expression analysis and also had increased levels of 5hmC in their promoters, Venn diagram showed that 98 genes had such patterns (Figure 2b). GO analysis of the 98 genes categorized them into different groups (Figure 2c). It is interesting that the genes belonging to cholesterol metabolic process represented the most statistically significant group (Figure 2c), which was also one of the prominently represented groups from RNA-Seq analysis (Additional file 5a). *INSIG1* (insulin induced gene 1), a major regulator of cholesterol biosynthesis [20,21], was inside this group (Figure 2a and c). INSIG1 sequesters SREBP in ER to inhibit cholesterol synthesis and promotes the degradation of HMG CoA reductase [20,21]. We further focused on the analysis of *INSIG1* gene since HIF-1α inhibits sterol regulatory element binding protein-1c (SREBP-1c) and suppresses excessive lipid accumulation [22,23]. Analysis of *INSIG1* RNA levels confirmed the activation of *INSIG1* expression by hypoxia and knockdown of TET1 decreased the expression of *INSIG1* under hypoxia in two different cell lines using quantitative real-time PCR analysis (Figure 2d and e and Additional file 6b and c).

### Demethylation of the *INSIG1* promoter regions by TET1

**Figure 3:**
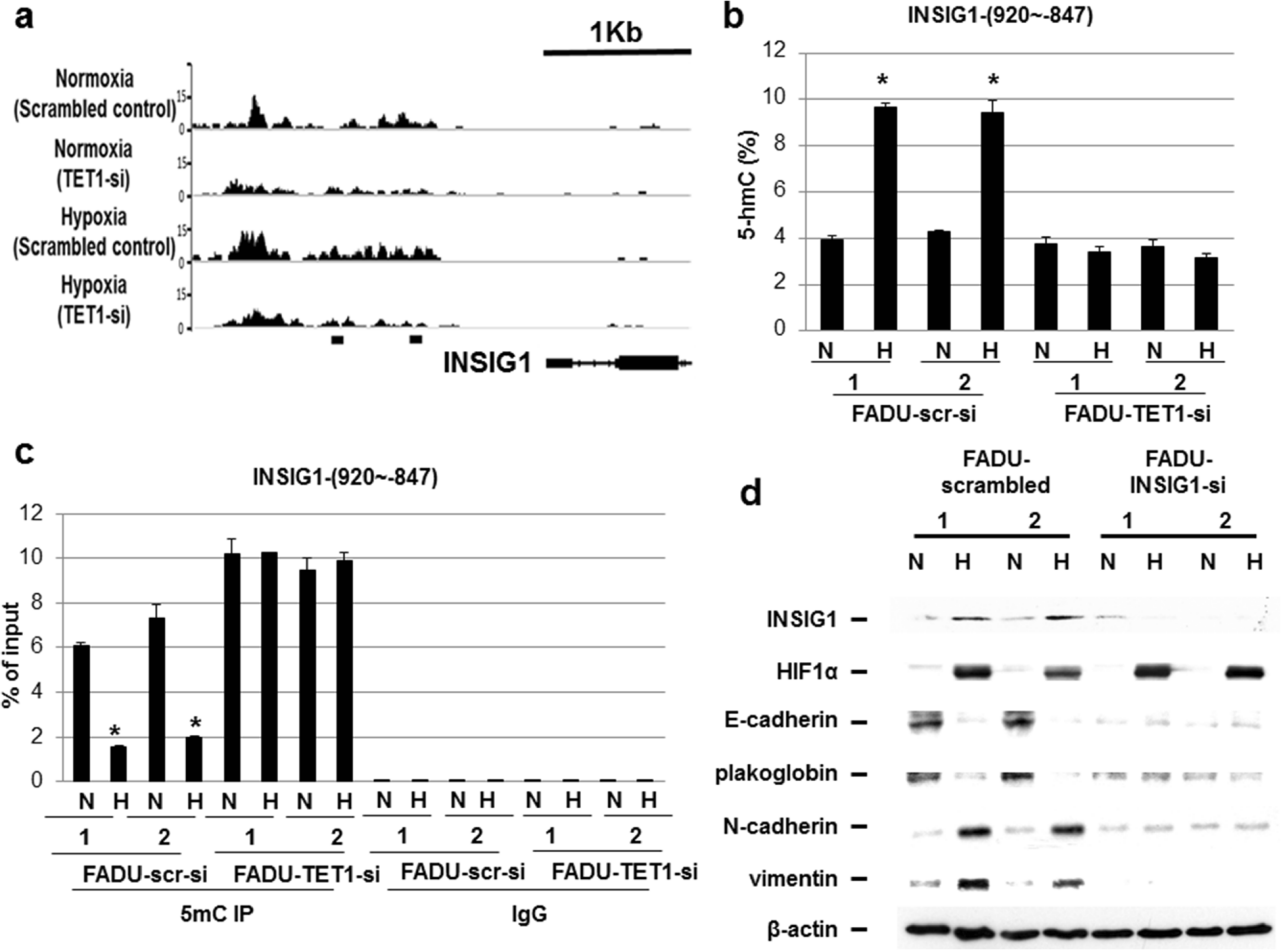
**Localization of 5hmC peaks in the** ***INSIG1*** **gene promoter by analysis of 5hmC sequencing, confirmation of the 5hmC peaks by hMeDIP and MedIP analysis, and knockdown of INSIG1 mitigated the process of hypoxia-induced epithelial-mesenchymal transition. (a)** Visualization of 5hmC signal in the *INSIG1* promoter. The y axis is normalized read counts. The regions analyzed for 5hmC and 5mC levels **(b,c)** were labeled as dark brackets. **(b)** Confirmation of increased 5hmC peaks in the promoter region of *INSIG1* gene using hMeDIP analysis. N; normoxia; H: hypoxia. **(c)** Confirmation of decreased 5mC peak in the promoter of *INSIG1* gene using MeDIP analysis. The asterisk (*) indicates statistical significance (*P* <0.05) between experimental and control clones. The FADU scrambled control clone under normoxia was chosen as the control condition in **(b)** and **(c)**. Error bars indicate standard deviations (s.d.) of duplicate analysis by PCR **(b,c)**. **(d)** Knockdown of INSIG1 mitigated hypoxia-induced epithelial-mesenchymal transition.

To correlate the RNA expression and 5hmC levels in the promoter region regulated by TET1, the group of cholesterol metabolic process genes including *INSIG1*, *FDPS* (farnesyl diphosphate synthase), *APOC1* (apolipoprotein C1), and *SQLE* (squalene epoxidase) were analyzed. There were increased 5hmC peaks in their promoters (Figure 3a and Additional file 7). Since the expression of *INSIG1* was confirmed (Figure 2d and e and Additional file 6b and c), we further analyzed the 5hmC peaks in the promoter of *INSIG1*. The 5hmC peaks in two different promoter regions of *INSIG1* gene were confirmed to be increased by hypoxia and decreased under TET1 knockdown in two different cell lines using hMeDIP assays (Figure 3b and Additional file 8). In addition, the levels of 5mC also decreased in the promoter region of *INSIG1* under hypoxia in two different cell lines using MeDIP assays (Figure 3c and Additional file 9). Finally, to test the role of INSIG1 in hypoxia-induced EMT, knockdown of INSIG1 was performed. The result showed that knockdown of INSIG1 abolished hypoxia-induced EMT in FADU and H1299 cells, mainly abolishing mesenchymal gene activation (Figure 3d and Additional file 10a). Increased *in vitro* migration and invasion activity in H1299 cells induced by hypoxia was abolished under INSIG1 knockdown (Additional file 10b). Further analysis showed that the abolishment of hypoxia-induced EMT by knockdown of INSIG1 was not mediated through repression of EMT regulators including Twist1 and Snail (Additional file 10c). All the above results indicate that INSIG1 plays a significant role in hypoxia-induced EMT, which is regulated by TET1.

### Regulation of glucose metabolism gene expression by TET1 or INSIG1

**Figure 4:**
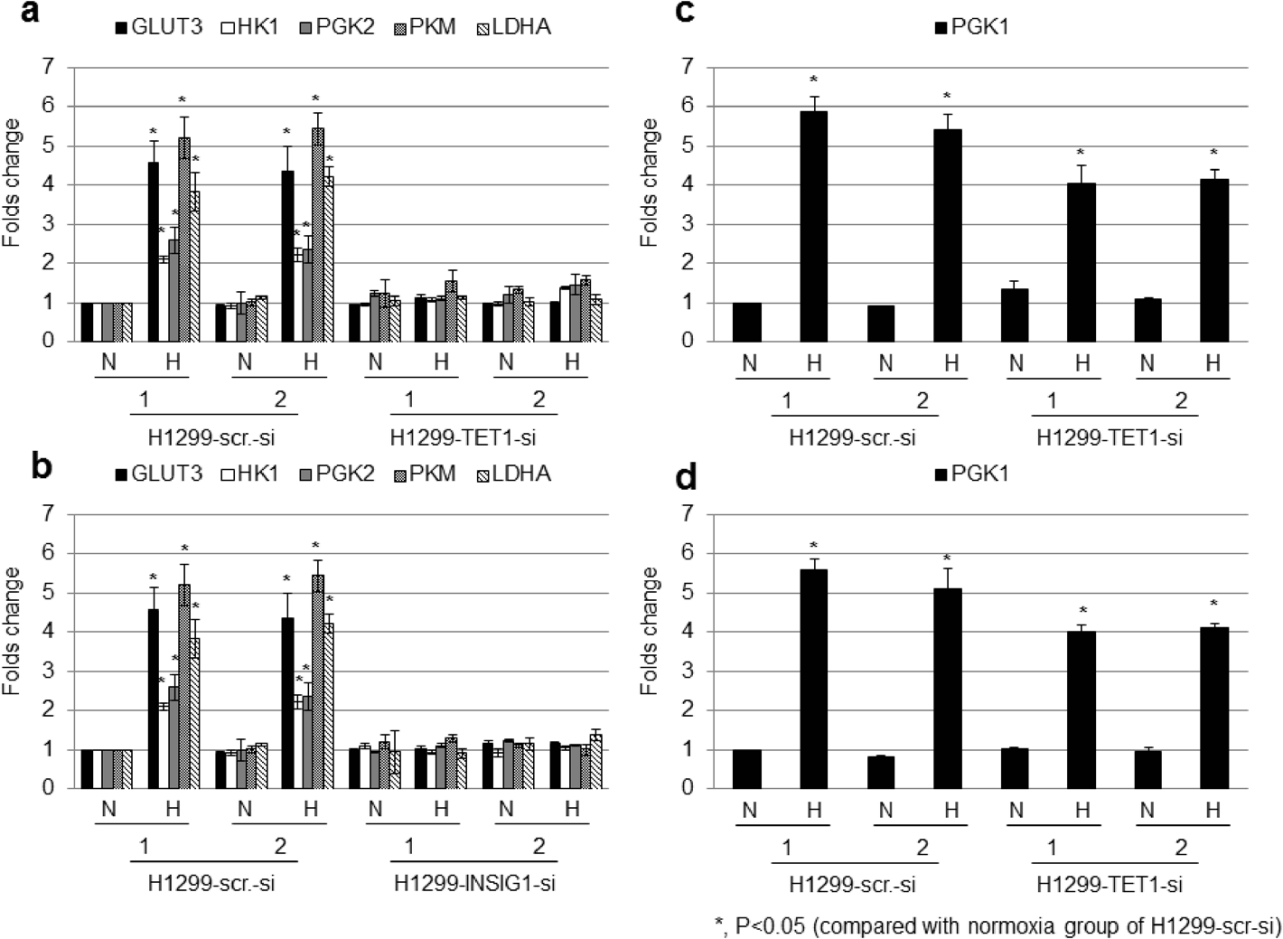
**TET1 and INSIG1 regulated expression of glucose metabolism genes induced by hypoxia in H1299 cells. (a,b)** Induction of *GLUT3*, *HK1*, *PGK2*, *PKM*, and *LDHA* expression by hypoxia, which were regulated by TET1 and INSIG1. **(c,d)** Hypoxia-induced *PGK1* expression was not regulated by TET1 or INSIG1. The asterisk (*) indicates statistical significance (*P* <0.05) between experimental (hypoxia) and control (normoxia) clones.

We tested whether TET1 or INSIG1 regulates glucose metabolism pathway to possibly contribute to hypoxia-induced Warburg effect [24,25]. The genes in the glucose metabolism pathway were tested for their expression under hypoxia and knockdown of TET1 or INSIG1. The results showed that the genes including glucose transporter 3 (*GLUT3*), hexokinase 1 (*HK1*), phosphoglycerate kinase 2 (*PGK2*), pyruvate kinase M (*PKM*), and lactate dehydrogenase A (*LDHA*) were induced by hypoxia and knockdown of TET1 or INSIG1 abolished or decreased their expression levels in two different cell lines (Figure 4a,b, and Additional file 11a,b). In contrast, phosphoglycerate kinase 1 (*PGK1*) was induced by hypoxia but did not respond to TET1 or INSIG1 knockdown (Figure 4c,d and Additional file 11c,d). These results indicate that TET1 and INSIG1 regulate the expression of hypoxia-induced glycolytic genes and may contribute to Warburg effect induced by hypoxia.

### TET1 acts as a transcription co-activator to regulate hypoxia-induced gene expression

**Figure 5:**
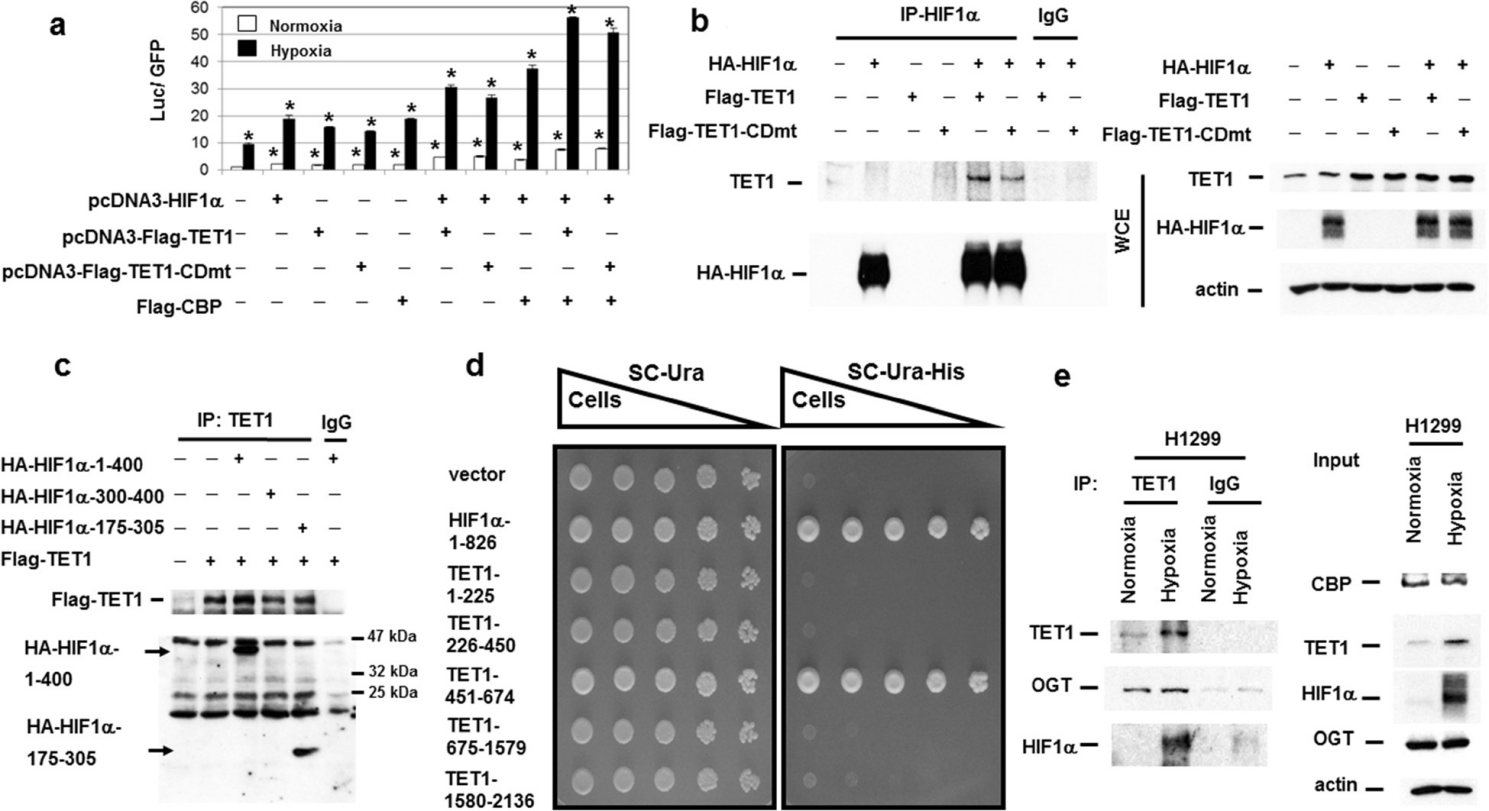
**TET1 served as a co-activator to activate hypoxia downstream target and TET1 interacted with HIF-1α through a specific domain together with mapping of the transactivation domain in TET1. (a)** Synergistic activation of the Twist1-driven reporter construct by co-transfecting HIF-1α, TET1 (wild-type or point mutant), and CBP. N: normoxia; H: hypoxia. The asterisk (*) indicates statistical significance (*P* <0.05) between experimental and control transfections. pXP2-Twist1 promoter-driven luciferase construct alone was used as the control transfection. Error bars indicate standard deviations (s.d.) of triplicate luciferase activity. **(b)** Co-immunoprecipitation assays showed the interaction between HIF-1α and TET1. The expression levels of transfected proteins (WCE: whole cell extracts) were shown on the right panel. **(c)** Mapping of the domain in HIF-1α that interacted with TET1. The HIF-1α-175-305 truncation mutant interacted with TET1 using the co-immunoprecipitation assays. **(d)** Yeast one hybrid assays showed that the transactivation activity of TET1 is mapped to the region of a.a. 451-674. The HIF-1α (a.a. 1-826) was used as a positive control. The yeasts transformed with different plasmids can survive in the Sc-Ura medium, whereas only the yeasts containing plasmids that can activate the HIS3 gene can survive in the selection medium of Sc-Ura-His. **(e)** Co-immunoprecipitation experiments showed that the anti-TET1 antibody pulled down both endogenous OGT and HIF-1α in H1299 cells.

Since there were TET1-regulated genes that did not show difference in the 5hmC levels in their promoters (Figure 2b), we speculated that TET1 may serve as a co-regulator together with HIFs to regulate gene expression. To test this hypothesis, reporter gene assays using HIF-1α, TET1, CBP (CREB binding protein), and *Twist1* promoter-driven reporter construct were performed in 293 T cells. Our previous results indicated that wild-type HIF-1α could be stably expressed in 293 T cells due to the overexpression of HIF-1α that is beyond the HIF-1α degradation activity under normoxia [6,11]. Indeed TET1 further enhanced the reporter gene activity similar to CBP (Figure 5a). In addition, the TET1 enzymatically inactive mutant (TET1-CDmt) also had similar activity to increase the ability of HIF-1α to promote *Twist1* promoter activity (Figure 5a) [26]. Similar results were shown using the *WDR5* promoter-driven reporter construct (Additional file 12a). In addition, using the *INSIG1* promoter-driven reporter gene, the ability of HIF-2α to promote its activity was further enhanced by either TET1 wild-type or TET1-CDmt protein (Additional file 12b and c). These results indicate that TET1 may serve as a co-activator to enhance the transactivation activity of either HIF-1α or HIF-2α.

For TET1 to be a co-activator of HIFs, there should be interaction between TET1 and HIFs. Co-immunoprecipitation assays were performed by co-transfecting HIF-1α and TET1 into 293 T cells. The results showed that the anti-HIF-1α antibody pulled down both HIF-1α and TET1 (Figure 5b). Reverse assays also showed that the anti-TET1 antibody pulled down TET1 and HIF-1α (Additional file 13a). HIF-1α also interacted with the enzymatically inactive TET1 (TET1-CDmt) by co-immunoprecipitation assays (Figure 5b and Additional file 13a). The interaction also occurred between HIF-2α and TET1 as well as between HIF-2α and TET1-CDmt (Additional file 13b and c). To further map the domain in HIF-1α that interacts with TET1, co-immunoprecipitation assays using various truncation mutants of HIF-1α and TET1 showed that the amino terminal domain (a.a. 1-400) of HIF-1α interacted with TET1 (Additional file 14). Further mapping experiments showed that the a.a. 175-305 region of HIF-1α interacted with TET1 (Figure 5c and Additional file 15a and b). To map the domain in TET1 that has transactivation activity, a yeast one hybrid assay was performed. The a.a. 451-674 region in TET1 displayed transactivation activity in contrast to the other regions that did not provide transactivation activity (Figure 5d). Further dissection of the transactivation domain showed that dividing this domain into two regions disrupted the transactivation activity (Additional file 15c). All the above results indicate that TET1 serves as a co-activator to interact with HIFs and activate the expression of hypoxia downstream targets.

Since O-linked N-glucosamine (O-GlcNAc) transferase (OGT) interacts with TET1 to modulate TET1 levels and regulate CpG island methylation [27,28], we tested whether OGT could also interact with HIF-1α and TET1 to regulate HIF-1α target gene expression. Co-immunoprecipitation experiments using an anti-TET1 antibody to pull down extracts from H1299 cells showed that both HIF-1α and OGT can be pulled down by an anti-TET1 antibody (Figure 5e), indicating that endogenous TET1 interacts with HIF-1α and OGT. However, only TET1 and CBP can be pulled down by an anti-HIF-1α antibody, but not OGT (Additional file 16a). This result suggested that there may not be direct interaction of HIF-1α with OGT. Other co-immunoprecipitation experiments showed that only TET1 can be pulled down by an anti-OGT antibody, but not HIF-1α or CBP (Additional file 16b). In addition, only HIF-1α can be pulled down by an anti-CBP antibody, but not OGT or TET1 (Additional file 16c). Co-expression of OGT with HIF-1α and CBP did not further increase the HIF-1α target gene expression using transient transfection assays (data not shown). These results indicate that TET1 may form different complexes with HIF-1α/CBP or OGT to regulate different target gene expression.

### Rescue of hypoxia-induced EMT in TET1 knockdown cells by a TET1 catalytically inactive mutant

**Figure 6:**
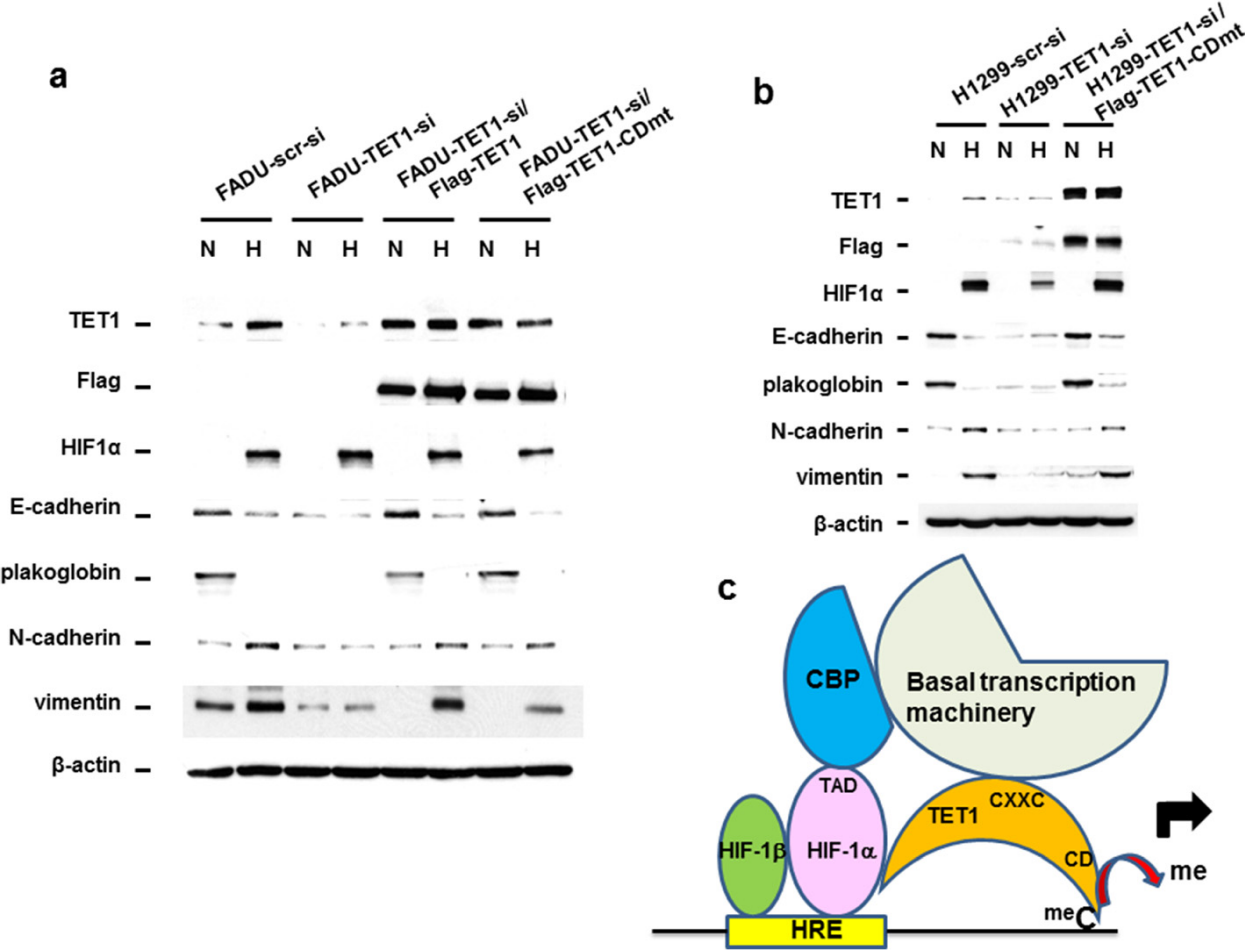
**Rescue of hypoxia-induced EMT in TET1 knockdown cells by a catalytically inactive TET1 mutant, and a model to depict the discovery of this report. (a,b)** Expression of the TET1-CDmt expression vector in FADU **(a)** or H1299 **(b)** cells with TET1 knockdown restored the phenotype of hypoxia-induced EMT. **(c)** The model of TET1 serving as a 5mC dioxygenase and transcription activator. Red arrow indicates the process of demethylation of 5mC by TET1. TAD: transactivation domain of HIF-1α; CXXC: CXXC domain of TET1 protein; CD: catalytic domain of TET1 protein; meC: 5-methylcytosine; me: methyl group; HRE: hypoxia-response element.

Since TET1 knockdown mitigated hypoxia-induced EMT and TET1 can act as a transcriptional co-activator without the enzymatic activity, we tested whether a TET1 catalytically inactive mutant could rescue hypoxia-induced EMT in TET1 knockdown cells. The TET1-CDmt expression vector was transfected into two different TET1 knockdown cell lines to test its ability to rescue hypoxia-induced EMT. The results showed that TET1-CDmt was capable of restoring hypoxia-induced EMT in two different TET1 knockdown cell lines (Figure 6a,b), indicating that the catalytic activity of TET1 is dispensable for hypoxia-induced EMT.

## Conclusions

This report demonstrated that hypoxia-induced TET1 uses another important mechanism by serving as a transcription co-activator to regulate hypoxia-responsive gene expression and EMT. It is interesting that INSIG1, the master regulator of cholesterol biosynthesis [20,21], is involved in the regulation of hypoxia-induced EMT, highlighting the novelty and importance of lipid metabolism in regulating EMT and metastasis. Due to the ability of INSIG1 to inhibit cholesterol biosynthesis [20,21] that may shun down lipid synthesis to favor glucose utilization similar to the role of AMPK that also inhibits cholesterol synthesis [29], the activation of INSIG1 expression through hypoxia-induced TET1 may also contributes to Warburg effect observed under hypoxia as supported by our results (Figure 4) [1,2]. It is interesting that INSIG1 did not regulate hypoxia-induced EMT through Twist1 or Snail, indicating that other EMT regulators or mechanisms (for example, chromatin modifiers) may be involved. Since we readily observed the regulation of various glucose metabolic genes by INSIG1, the cross talk between metabolism and other mechanisms may explain the role of INISG1 in hypoxia-induced EMT.

Our results show that TET1 serves as a 5mC-specific dioxygenase to mediate DNA demethylation of promoters [10,11], while it also simultaneously interacts with transcription factors as a co-activator to promote gene transcription. This mechanism is a unique model of gene transcription and a model is presented (Figure 6c). TET1 therefore represents a new class of co-activator compared to the recent demonstration of PKM2 (pyruvate kinase M2) as a HIF-1α co-activator [30]. The domain in TET1 that confers transactivation activity is mapped (a.a. 451-674) by co-immunoprecipitation and yeast one-hybrid assays. It is interesting that this region contains the CXXC domain that is located in the TET enzymes [31]. In contrast to the recruitment of TET1 by NANOG that requires the catalytic activity of TET1 to establish pluripotency [32], synergistic activation by TET1 and HIFs is independent of its enzymatic activity and supports that TET1 is a bona fide transcription co-activator. Our results demonstrate that TET1 plays a crucial role in the regulation of hypoxia-responsive gene expression by acting as a transcriptional co-activator since the catalytically inactive TET1 could rescue hypoxia-induced EMT in TET1 knockdown cells. It is possible that TET1 also serves as a co-repressor since Tet1 has dual functions on gene transcription and also interacts with SIN3A [15,33]. Although TET1 also interacts with OGT to regulate gene expression [27,28], our results showed that TET1/OGT may form a different complex from the HIF-1α/CBP/TET1 complex. It will be important to search for other transcription factors that can interact with TET1 to regulate gene expression. Our results further expand the role of TET1 beyond epigenetic regulation of gene expression.

## Materials and methods

### Cell culture

The cell lines used were described [6,11] including the human hypopharyngeal squamous carcinoma cell line FADU, human tongue squamous carcinoma cell line SAS, and lung cancer cell lines H1299 and A549 (obtained from ATCC). Human embryonic kidney 293 T cell line was used in transient transfection experiments. SNU398 is a human hepatoma cell line obtained from Dr. YS Jou (Academia Sinica, Taiwan).

### Protein extraction, western blot analysis, RNA extraction, quantitative real-time PCR, *in vitro* migration/invasion assay 

For extraction of proteins from cell lines, cell lysis buffer (50 mM Tris, pH 7.5, 150 mM NaCl, 0.5% sodium deoxycholate, 1% NP-40, 0.1% SDS) containing protease inhibitors was used. Cell lysates were clarified by centrifugation at 13,000 rpm, 4°C for 10 min. The protein content was determined by Bradford method (Bio-Rad Laboratories, Hercules, CA, USA). For western blot analysis, 50 μg to 100 μg protein extracts from each clone were loaded to 10% SDS-PAGE gels and transferred to nitrocellulose filters. The filters were probed with different antibodies (Additional file 17), and an anti-ß-actin antibody was selected as a loading control. Signals were developed using an ECL chemiluminescence kit (Millipore Corporation, Billerica, MA, USA). Total RNA was isolated using Trizol reagent (Life Technologies Corporation, Carlsbad, CA, USA) according to manufacturer’s recommendations. Single-stranded cDNA was synthesized by the RevertAid™ First Strand cDNA Synthesis Kit (Fermentas International, Inc., Burlington, Canada). Real-time PCR was performed on a StepOnePlus™ Real-Time PCR System (ABI: Applied Biosystems, Foster City, CA USA) according to the manufacturer’s instructions. The 2-ΔΔCt method of relative quantification was used to estimate the copy number of gene expression, and 18S was selected as an internal control. The oligonucleotides used for real-time PCR were shown in Additional file 18. For *in vitro* migration/invasion assay, eight-μm pore size Boyden chamber was used. Cells (4 × 10^4^) in 0.5% serum-containing RPMI were plated in the upper chamber and 15% fetal bovine serum was added to RPMI 1640 in the lower chamber as a chemo-attractant. For invasion assay, the upper side of the filter was covered with Matrigel (BD Biosciences, San Jose, CA, USA) (1:2 dilution with RPMI). After 12 h for migration assay or 24 h for invasion assay, cells on the upper side of the filter were removed, and cells that remained adherent to the underside of membrane were fixed in 4% formaldehyde and stained with Hoechst 33342 dye. The number of migrated cells was counted using a fluorescence microscope. Ten contiguous fields of each sample were examined using a 40× objective to obtain a representative number of cells which migrated/invaded across the membrane.

### Plasmids, transfection, and luciferase assays

The plasmids used and transfection methods were described using calcium phosphate or lipofection transfection method [6,11]. The expression vectors of HIF-1α, HIF-2α, CBP, Twist1 promoter-driven luciferase construct, and WDR5 promoter-driven luciferase construct were described [6,11]. The *TET1* and *INSIG1* promoter regions were cloned and the reporter constructs were shown in Additional file 2. The oligonucleotides used for plasmis constructions were shown in Additional file 19. The reporter constructs were co-transfected into 293 T cells with different expression vectors and an internal control plasmid. Luciferase assays were performed using the same amount of cell extracts and corrected for transfection efficiency using an internal control (GFP) [6,11].

### Quantitative chromatin immunoprecipitation (Chip)

ChIP assay was performed as described [6]. Briefly, cells were cross-linked with 1% formaldehyde for 10 min and stopped by adding glycine to a final concentration of 0.125 M. Fixed cells were washed twice with TBS (20 mM Tris, pH 7.5, 150 mM NaCl) and harvested in 5 mL of SDS buffer (50 mM Tris, pH 8.0, 0.5% SDS, 100 mM NaCL, 5 mM EDTA, and protease inhibitors). Cells were pelleted by centrifugation and suspended in 2 mL of IP buffer (100 mM Tris, pH 8.6, 0.3% SDS, 1.7% Triton X-100, 5 mM EDTA). Cells were sonicated with a 0.25-inch diameter probe for 15 s twice using an MSE-soniprep 1500 sonicator (setting 18). For each immunoprecipitation, 1 mL of lysate was precleared by adding 50 μL of blocked protein A beads (50% protein A-Sepharose, Amersham Biosciences; 0.5 mg/mL bovine serum albumin, 0.2 mg/mL salmon sperm DNA) at 4°C for 1 h. Samples were spun, and the supernatants were incubated at 4°C for overnight with no antibody, IgG, anti-HIF-2α antibody. Immune complexes were recovered by adding 50 μL of blocked protein A beads and incubated overnight at 4°C. Beads were successively washed with: (1) mixed micelle buffer (20 mM Tris, pH 8.1, 150 mM NaCl, 5 mM EDTA, 5% w/v sucrose, 1% Triton X-100, 0.2% SDS); (2) buffer 500 (50 mM Hepes, pH 7.5, 0.1% w/v deoxycholic acid, 1% Triton X-100, 500 mM NaCl, 1 mM EDTA); (3) LiCl detergent wash buffer (10 mM Tris, pH 8,0.5% deoxycholic acid, 0.5% Nonidet P-40, 250 mM LiCl, 1 mM EDTA); and (4) TE buffer (10 mM Tris, 1 mM EDTA) and then eluted with 1% SDS and 0.1 M NaHCO_3_. Twenty milliliters of 5 M NaCl was added to the elutes, and the mixture was incubated at 65°C for 5 h to reverse the cross-linking. After digestion with proteinase K, the solution was phenol/chloroform-extracted and ethanol-precipitated. For qChIP analysis [11], DNA fragments were resuspended in 400 μL of water and 5 μL was used by real-time PCR. Each sample was calculated as the percentage of input sample. The sequences of PCR regions and primers used in ChIP assay are listed (Additional file 20). The antibodies used are listed (Additional file 17).

### Immunofluorescence staining 

For immunofluorescence staining [6,11], cells on glass coverslips or chamber slides were fixed with 4% paraformaldehyde and permeabilized with 0.5% Triton X-100. After washing three times for 10 min with PBS, fixed cells or slides were blocked with blocking buffer (PBS, 0.1% Tween-20, with 3% goat serum) for 1 h and incubated with primary antibody diluted in blocking buffer overnight at 4°C. After washing three times with PBS for 10 min, the fixed cells were treated with the appropriate secondary antibody (fluorescein isothiocyanate (FITC)-conjugated anti-mouse IgG or rhodamine-conjugated anti-rabbit IgG) (Sigma) that was diluted in blocking buffer for 1 h at room temperature. Finally, the fixed cells were washed three times for 10 min with TBS, and their nuclei were counterstained, mounted, and observed by using fluorescence microscope or confocal microscope.

#### Lentiviral infection

Lentivirus containing short hairpin RNAs (shRNAs) expressed in a lentiviral vector (pLKO.1-puro) were generated in 293 T cells as previously described [11]. Packaging plasmid pCMVΔR8.91 was obtained from SC Teng (National Taiwan University, Taiwan). Various pLKO plasmids to knockdown HIF-1α, HIF-2α, TET1, INSIG1, and scrambled control were provided by National RNAi Core Facility of Academia Sinica, Taipei, Taiwan. For lentivirus production, 293 T cells were transfected with 15 μg pLKO.1-puro lentiviral vectors expressing different shRNAs along with 1.5 μg of envelope plasmid pMD.G and 15 μg of packaging plasmid pCMVΔR8.91. Virus was collected 48 h after transfection. To prepare various knockdown cells, FADU or H1299 cells were infected with lentivirus for 24 h, and used for various assays after 4 to 5 days. The sequence of the lentiviral siRNA vectors were shown in Additional file 21.

### RNA-Seq data analysis

The RNA-Seq libraries were prepared according to the standard Illumina protocol with the mRNAseq Illumina TruSeq and were sequenced using Illumina Hiseq2000 to obtain 100 bp paired-end reads. The reads were aligned to the hg19 reference assembly with the TopHat/Cufflinks [34] alignment package using Ensembl annotations. Transcript abundance was measured in fragments per kb of exon per million fragments mapped (FPKM).

### 5hmC chemical labeling

5hmC labeling reactions were performed according to the previous protocol [35]. Briefly, sonicated genomic DNA (average 400 bp, 500 ng/μL) was incubated with 50 mM HEPES buffer (pH 7.9), 25 mM MgCl_2_, 100 mM UDP-6-N_3_-Glc, and 2 mM βGT for 1 h at 37°C. The labeled DNA was purified by the QIAquick Nucleotide Removal kit (QIAGEN) and eluted in H_2_O. The click chemistry was performed with the addition of 150 mM of disulfide-biotin, and the mixture was incubated for 2 h at 37°C. The labeled DNA fragments were then purified by the QIAquick Nucleotide Removal kit (QIAGEN) and enriched by Dynabeads Streptavidin C1 (Invitrogen), and subsequently released by DTT treatment. The enriched DNA fragments were first purified by Micro Bio-Spin 6 spin columns (Bio-Rad) followed by MinElute PCR Purification Kit (QIAGEN).

### 5hmC-Seq data analysis

All the 5hmc-enriched DNAs were sequenced using Illumina Hiseq2000 containing more than 2.5 × 10^7^, 50 bp single-end reads per sample. Raw sequence reads were mapped onto the reference human genome (NCBI Build UCSC hg19) using the Bowtie v0.12.7 algorithm (-m 1 -v 3 --best --strata) [36]. Unique and monoclonal reads were used for further analysis. The distribution of 5hmC reads at promoters or in gene body regions were analyzed by PAVIS [37] (Additional file 6a). Further analysis was done using Bioconductor using packages ChIPpeakAnno, biomaRt, MEDIPS and in-house scripts. The genes with 5hmC peaks at promoter region (approximately -3000 to 0 bp upstream of the transcription start site) were chosen for further analysis.

### Selection of overlapping groups of genes from RNA-Seq and 5hmC-Seq data followed by Gene Ontology (GO) analysis

The genes that have differential 5hmC-enriched promoter regions from 5hmC-Seq analysis and are differentially regulated from RNA-Seq analysis are selected for functional enrichment analysis. For 5hmC-Seq data, the criteria for genes with 5hmC-enriched promoter regions are: the peak levels of Hypoxia (scrambled control) >4X Hypoxia (TET1-si), Hypoxia (scrambled control) >2X Normoxia (scrambled control), and Normoxia (scrambled control) >2X Normoxia (TET1-si). The differentially expressed genes from RNA-Seq are identified by the criteria: Hypoxia (scrambled control) >2X Hypoxia (TET1-si), Hypoxia (scrambled control) > Normoxia (scrambled control), and Normoxia (scrambled control) > or = Normoxia (TET1-si). The 98 genes that were chosen through the overlapping criteria by 5hmC-Seq and RNA-Seq analyses were further analyzed by GO-based functional enrichment analysis. GO-based functional enrichment analysis (Fisher’s Exact test) was used to measure the gene-enrichment in annotation terms for selected genes. The genes in the GO terms that passed the criteria of *P* value <0.01 and at least two genes in each GO term were considered for further analysis.

### Detection of 5hmC levels within specific gene locus (hMeDIP)

Genomic DNA was prepared using a genomic DNA extraction kit (QIAGEN, Germany). Detection of 5-hydroxymethylcytosine (5hmC) within specific gene locus was performed by using EpiJET 5-hmC Analysis Kit (Thermo Scientific, USA). The DNA was glucosylated and digested with the Glc-5-hmC sensitive restriction endonuclease Epi MspI. Glucosylation/digestion levels were analyzed by qPCR. The sequences of oligonucleotides used for hMeDIP were shown in Additional file 22.

### Methylated DNA immunoprecipitation (MeDIP)

Genomic DNA was prepared using a genomic DNA extraction kit (QIAGEN, Germany) and sonicated with Bioruptor (Diagenode Inc., USA) to produce random fragments ranging in mean size from 300 to 1,000 bp. Two micrograms of fragmented DNA was denatured for 10 min at 95°C and immunoprecipitated for 16 h at 4°C with 5 μL of 5-methylcytidine antibody (Eurogentec, Germany) in a final volume of 250 μL IP buffer (20 mM Tri-HCl pH 7.5, 150 mM NaCl, 1 mM EDTA, 1 mM EGTA, 2.5 mM sodium pyrophosphate, 1 mM β-glycerophosphate, 0.05% Triton X-100). The mixture was incubated with 50 μL protein A beads for another 4 h at 4°C and washed three times with 1 mL of IP buffer. Beads were resuspended with 500 μL digestion buffer (50 mM Tris (pH 8.0), 10 mM EDTA, 1% SDS) containing 10 μL proteinase K (20 mg/mL stock) overnight at 65°C. DNA was phenol/chloroform-extracted, ethanol-precipitated, and resuspended in 200 μL of water for real-time PCR analysis. The sequences of oligonucleotides used for MeDIP were shown in Additional file 22.

### Yeast strains, transformations, and media

PJ69-4A (*MATa trp1-901 leu2-3,112 ura3-52 his3-200 gal4Δ gal80Δ LYS2∷GAL1-HIS3 GAL2-ADE2 met2∷GAL7-lacZ*) was used as the yeast strain. Media was prepared as described [38]. All yeast transformations were conducted using the high efficiency method described [39].

### Co-immunoprecipitation assays

Co-immunoprecipitation was performed by incubating antibody with 500 μL of whole cell extracts in IP buffer (20 mM Tri-HCl pH 7.5, 150 mM NaCl, 1 mM EDTA, 1 mM EGTA, 1% Triton X-100, 2.5 mM sodium pyrophosphate, 1 mM β-glycerophosphate, and protease inhibitor) with 50 μL of protein A/G mix magnetic beads (Millipore) for 4 h at 4°C. The immunoprecipitates were washed several times with IP buffer to removing any non-bound proteins. Finally, components of the bound immune complex are eluted, and then analyzed by western blot detection to verify the identity of proteins.

### Measurement of transcriptional activation strength

The activation strength was measured by two different assays which measure the expression of the reporter genes: *HIS3*, which encodes imidazoleglycerol-phosphate dehydratase and catalyzes the sixth step in yeast histidine biosynthesis. The pGBDU constructs containing HIF-1α, TET1, or TET1 truncation mutants were generated according to the length and restriction sites mentioned in Additional file 3. Plasmids were transformed into the yeast strain PJ69-4A [40]. Transformed clones were selected on SC-Ura plates and confirmed for HIS3 expression on SC-His plates. Five to 10 freshly transformed colonies were mixed and spotted in five-fold serial dilutions onto plates. Plates were kept at 30°C until colonies formed.

### Statistical analysis

The independent Student’s *t*-test was used to compare the continuous variables between two groups, and the *χ*^2^ test was applied for comparison of dichotomous variables. The control groups of all the statistical analyses were specified in the figure legends. The level of statistical significance was set at 0.05 for all tests.

### Accession number

Sequencing data have been deposited to the Gene Expression Omnibus (GEO) under accession number GSE59990.

## Additional files

Additional file 1: Figure S1. Activation of *TET1* expression by hypoxia in various cell lines, knockdown of HIF-2α abolished the activation of TET1 by hypoxia, and demonstration of HIF-2α binding to the promoter regions of *TET1* and *WDR5* genes. Additional file 2: Table S1. Information of plasmids for luciferase assay. Additional file 3: Figure S2. Immunofluorescence staining of E-cadherin and vimentin, and *in vitro* migration and invasion activity of H1299 cell lines with scrambled or TET1 knockdown under normoxia or hypoxia. Additional file 4: Figure S3. Knockdown of TET1 mitigated hypoxia-induced epithelial-mesenchymal transition in FADU cells, immunofluorescence staining of E-cadherin and vimentin, and *in vitro* migration and invasion activity of FADU cell lines with scrambled or TET1 knockdown under normoxia or hypoxia. Additional file 5: Figure S4. Gene Ontology analysis of the groups of genes that showed differential expression from RNA-Seq analysis and the global distribution of 5hmC peaks in FADU cells with scrambled or *TET1* knockdown under normoxia or hypoxia. Additional file 6: Figure S5. The percentage of distribution of 5hmC peaks, activation of *INSIG1* by hypoxia in FADU cells, and TET1 knockdown abolished *INSIG1* expression induced by hypoxia. Additional file 7: Figure S6. Localization of 5hmC peaks in the *FDPS* (a), *APOC1* (b), and *SQLE* (c) gene promoters by analysis of 5hmC sequencing. Additional file 8: Figure S7. 5hmC levels in the different promoter regions of *INSIG1* gene in H1299 cells or FADU cells under normoxia or hypoxia. Additional file 9: Figure S8. The 5-methylcytosine (5mC) levels in the promoter regions of *INSIG1* gene in FADU cells or H1299 cells under normoxia or hypoxia. Additional file 10: Figure S9. Knockdown of INSIG1 mitigated hypoxia-induced epithelial-mesenchymal transition in H1299 cells, and the levels of Twist1 or Snail levels were not affected by INSIG1 knockdown in FADU cells. Additional file 11: Figure S10. Real-time PCR analysis of the expression of various glycolytic enzymes (normoxia vs. hypoxia) under TET1 or INSIG1 knockdown. Additional file 12: Figure S11. Synergistic activation of *WDR5* or *INSIG1* promoter by HIF-2α, TET1 (wild-type or point mutant), and CBP under normoxia or hypoxia. Additional file 13: Figure S12. Co-immunoprecipitation experiments showed the interaction between TET1 and HIF-1α or HIF-2α. Additional file 14: Figure S13. Mapping of the domain in HIF-1α that interacted with TET1. Additional file 15: Figure S14. Further mapping of the domain in HIF-1α that interacted with TET1. Additional file 16: Figure S15. Various co-immunoprecipitation experiments to check the interaction between different proteins. Additional file 17: Table S2. List of proteins tested by antibodies and characteristics of the corresponding antibodies. Additional file 18: Table S3. Sequence of the oligonucleotides for real-time PCR. Additional file 19: Table S4. Sequence of the oligonucleotides for construct-making. Additional file 20: Table S5. Sequence of the oligonucleotides for ChIP analysis. Additional file 21: Table S6. Sequence of the lentiviral siRNA vectors. Additional file 22: Table S7. Sequence of the oligonucleotides for 5hmc assay and MeDIP.
