# Supplementary material for: TET1 regulates hypoxia-induced epithelial-mesenchymal transition by acting as a co-activator
Source: Genome Biol. 2014 Dec 3;15(12):513. doi: 10.1186/s13059-014-0513-0 (PMC4253621; doi:10.1186/s13059-014-0513-0)

**Additional file 1: Figure S1. Activation of *TET1* expression by hypoxia in various cell lines, knockdown of HIF-2α abolished the activation of TET1 by hypoxia, and demonstration of HIF-2α binding to the promoter regions of *TET1* and *WDR5* genes.** **(a)** Real-time PCR analysis showed the activation of *TET1* expression in five different cell lines. **(b, c)** Knockdown of HIF-2α abolished the expression of TET1 using real-time PCR and western blot analysis. **(d)** Chromatin immunoprecipitation assays showed the binding of HIF-2α to the promoter regions of *TET1* and *WDR5* genes. The asterisk (*) indicates statistical significance (*P* <0.05) between experimental and control clones. The control used for real time PCR analysis was the various cell lines under normoxia (N) (a), and H1299 cells or H1299-TET1-si or H1299-HIF-2α-si under nomoxia (b). The control used for ChIP assay was the FADU cells under normoxia (d). Error bars indicate standard deviations (s.d.) of duplicate mRNA levels by real-time PCR analysis (a, b) or ChIP analysis (d).


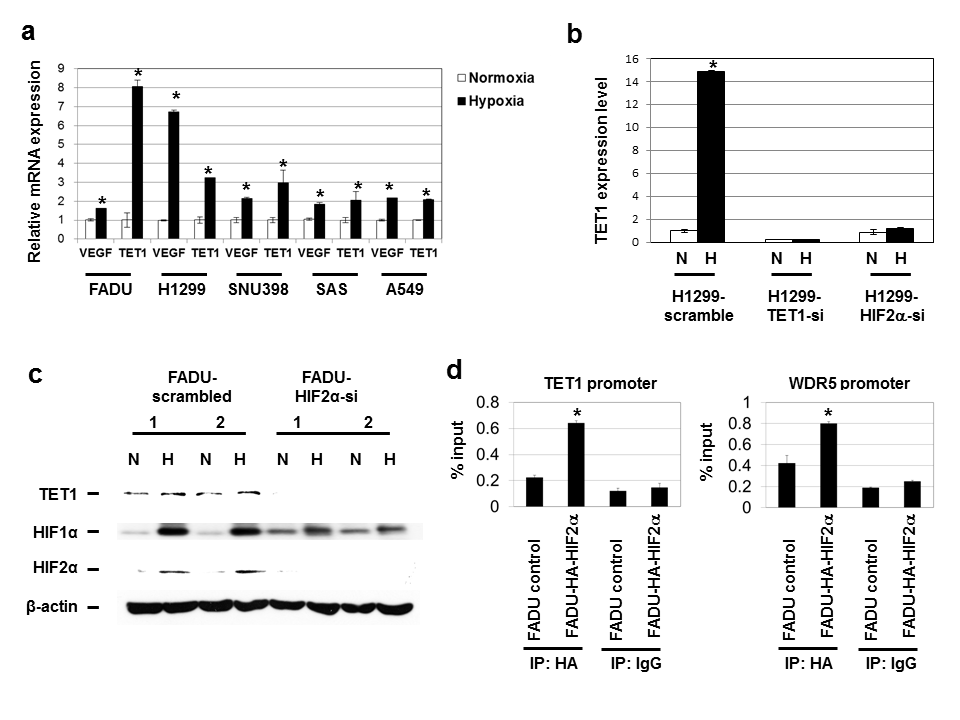

Supplement: Additional file 1: Figure S1. — Activation of TET1 expression by hypoxia in various cell lines, knockdown of HIF-2α abolished the activation of TET1 by hypoxia, and demonstration of HIF-2α binding to the promoter regions of TET1 and WDR5 genes. [file 13059_2014_513_MOESM1_ESM.doc]
