# Supplementary material for: TET1 regulates hypoxia-induced epithelial-mesenchymal transition by acting as a co-activator
Source: Genome Biol. 2014 Dec 3;15(12):513. doi: 10.1186/s13059-014-0513-0 (PMC4253621; doi:10.1186/s13059-014-0513-0)
Supplement: Additional file 2: Table S1. — Information of plasmids for luciferase assay. [file 13059_2014_513_MOESM2_ESM.doc]

**Additional file 2: Table S1. Information of plasmids for luciferase assay**

| **Plasmid** | **DNA origin** | **Human embryonic kidney 293T cells** |
| --- | --- | --- |
| pXP2-TET1-381 | Proximal *TET1* promoter sequence for promoter activity assay (-381 to +17 bp from TSS) | tcatctggtttctctctttggtgttgagggcggttttccgttgctggcgacctagacattgctcaatggtgtcatctctgacctggcaccttcttcatacatttataaacatgcaatttagagatcctaaaaactttttctagagctcccctgggaaaaaagttaatgacaaaatcttgacacctctctacgtcttcttcggagacagagaagttgagagaggtggagaacgagggggagggggagggggtcgagagggagtcgaggagggattccagctccagtttgggtaaatccagctcgcgttttgtctctcgctcaactgtgcagggtccagcgaaggcagagccccagcttcactccctgaggtctgtcctggggagacactgctgctccgg |
|  | Primers used in promoter cloning | F: GAACAAAGCTTTCATCTGGTTTCTCTCTTTGG |
|  |  | R: AGGTCAGATCTCCGGAGCAGCAGTGTCT |
| pXP2-TET1-158 | Proximal *TET1* promoter sequence for promoter activity assay (-158 to +17 bp from TSS) | ttggagaacgagggggagggggagggggtcgagagggagtcgaggagggattccagctccagtttgggtaaatccagctcgcgttttgtctctcgctcaactgtgcagggtccagcgaaggcagagccccagcttcactccctgaggtctgtcctggggagacactgctgctccgg |
|  | Primers used in promoter cloning | F: AAGCTTTGGAGAACGAGGGGGAG |
|  |  | R: AGGTCAGATCTCCGGAGCAGCAGTGTCT |
| pXP2-TET1-91 | Proximal *TET1* promoter sequence for promoter activity assay (-91 to +17 bp from TSS) | taaatccagctcgcgttttgtctctcgctcaactgtgcagggtccagcgaaggcagagccccagcttcactccctgaggtctgtcctggggagacactgctgctccgg |
|  | Primers used in promoter cloning | F: CAGTTAAGCTTAATCCAGCTCGCGT |
|  |  | R: AGGTCAGATCTCCGGAGCAGCAGTGTCT |
| pXP2-INSIG1-190 | Proximal *INSIG1* promoter sequence for promoter activity assay (-190 to +1 bp from TSS) | cccgctcgggcccgcgtgccccgcagccgccacgcgccccgcagtccgtgccgaggctcccgcccgagtgcgcgcgggccggcggtggcgtgtgcgcacgtcatatgggccgccggcctcccattggttgcggccgcctcaccagaccgcgtgcggacgggctcgcgggcggggcggggcaagctcaggcc |
|  | Primers used in promoter cloning | F: CCCAAGCTTGGCCAGTCCCGCTCGGGC |
|  |  | R: GAAGATCTGCCTGAGCTTGCCCCGCCCC |
