# Supplementary material for: TET1 regulates hypoxia-induced epithelial-mesenchymal transition by acting as a co-activator
Source: Genome Biol. 2014 Dec 3;15(12):513. doi: 10.1186/s13059-014-0513-0 (PMC4253621; doi:10.1186/s13059-014-0513-0)

**Additional file 3: Figure S2. Immunofluorescence staining of E-cadherin and vimentin, and *in vitro* migration and invasion activity of H1299 cell lines with scrambled or TET1 knockdown under normoxia or hypoxia.** **(a)** Immunofluorescence staining of E-cadherin (green) and vimentin (red) in H1299 cells with scrambled or TET1 knockdown under normoxia (N) or hypoxia (H). **(b)** *In vitro* migration and invasion activity of H1299 cells with scrambled or TET1 knockdown under normoxia (N) or hypoxia (H). The asterisk (*) indicates statistical significance (*P* <0.05) between experimental and control clones. The control used was the first H1299 scrambled control clone under normoxia (either migration or invasion). Error bars indicate standard deviations (s.d.) of triplicate numbers counted from either migration or invasion.


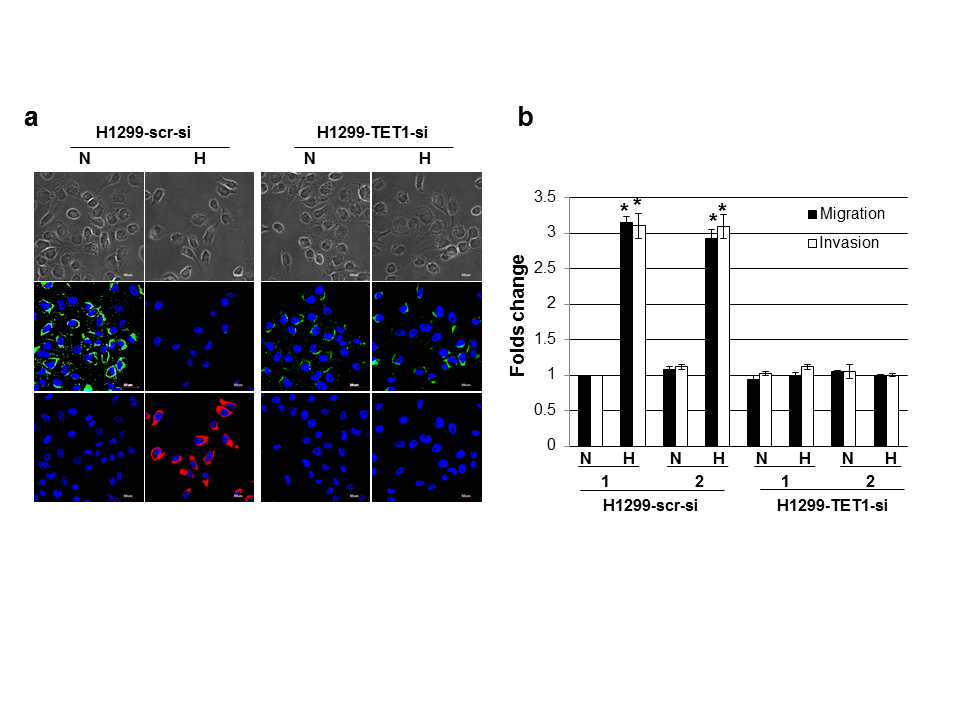

Supplement: Additional file 3: Figure S2. — Immunofluorescence staining of E-cadherin and vimentin, and in vitro migration and invasion activity of H1299 cell lines with scrambled or TET1 knockdown under normoxia or hypoxia. [file 13059_2014_513_MOESM3_ESM.doc]
