# Supplementary material for: TET1 regulates hypoxia-induced epithelial-mesenchymal transition by acting as a co-activator
Source: Genome Biol. 2014 Dec 3;15(12):513. doi: 10.1186/s13059-014-0513-0 (PMC4253621; doi:10.1186/s13059-014-0513-0)

**Additional file 5: Figure S4.** **Gene Ontology analysis of the groups of genes that showed differential expression from RNA-Seq analysis and the global distribution of 5hmC peaks in FADU cells with scrambled or *TET1* knockdown under normoxia or hypoxia. (a)** Ten groups of genes that had differential expression between normoxia and hypoxia from GO analysis were shown. **(b)** The global distribution of 5hmC peaks with regards to their positions in promoter regions, transcription start sites (TSSs), gene bodies, and so on (x axis). The y axis indicates normalized Tag density.


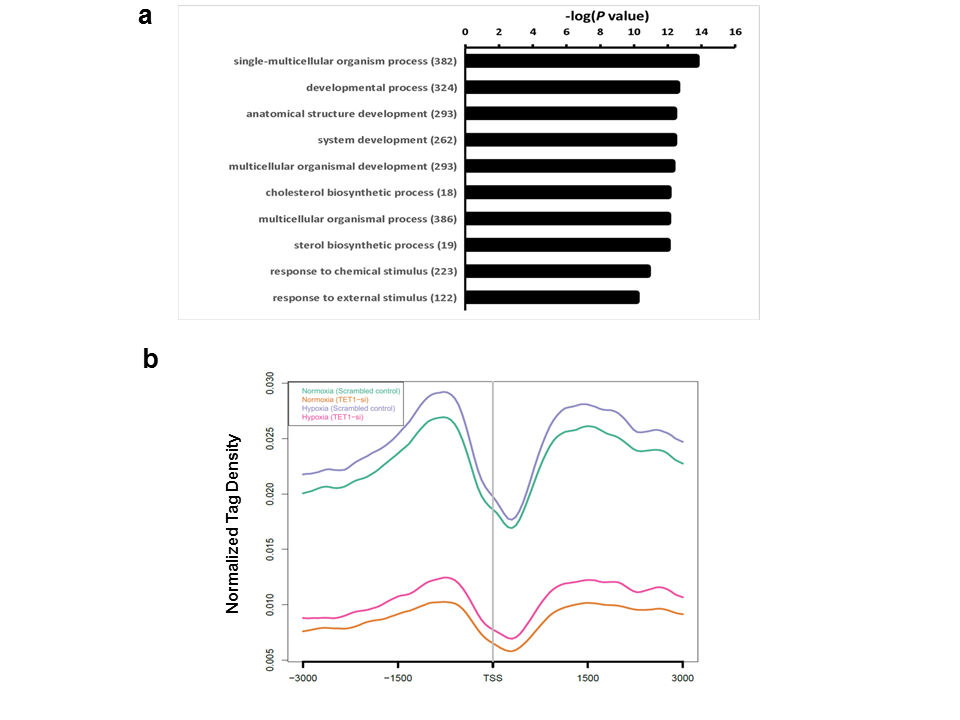

Supplement: Additional file 5: Figure S4. — Gene Ontology analysis of the groups of genes that showed differential expression from RNA-Seq analysis and the global distribution of 5hmC peaks in FADU cells with scrambled or TET1 knockdown under normoxia or hypoxia. [file 13059_2014_513_MOESM5_ESM.doc]
