# Supplementary material for: TET1 regulates hypoxia-induced epithelial-mesenchymal transition by acting as a co-activator
Source: Genome Biol. 2014 Dec 3;15(12):513. doi: 10.1186/s13059-014-0513-0 (PMC4253621; doi:10.1186/s13059-014-0513-0)

**Additional file 6: Figure S5. The percentage of distribution of 5hmC peaks, activation of *INSIG1* by hypoxia in FADU cells, and TET1 knockdown abolished *INSIG1* expression induced by hypoxia. (a)** The percentage of distribution of 5hmC peaks with regards to promoter regions (upstream), 5’UTR, exons, introns, 3’UTR, downstream, and other positions in FADU cells with scrambled or TET1 knockdown under normoxia or hypoxia***.* (b)** Activation of *INSIG1* expression by hypoxia in FADU cells using real-time PCR analysis. **(c)** Knockdown of *TET1* abolished the activation of *INSIG1* expression induced by hypoxia in FADU cells using real-time PCR analysis. The asterisk (*) indicates statistical significance (*P* <0.05) between experimental and control clones. The FADU vector (normal/ parental FADU) or FADU scrambled control clone under normoxia was chosen as the control condition in (b, c). Error bars indicate standard deviations (s.d.) of duplicate mRNA levels by real-time PCR analysis (b, c).


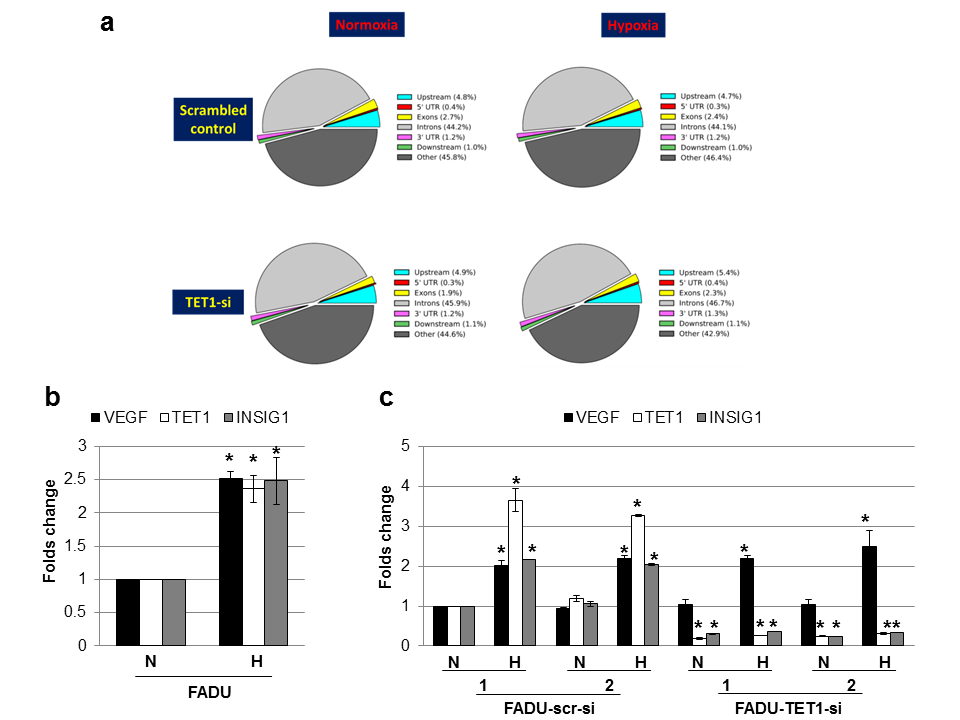

Supplement: Additional file 6: Figure S5. — The percentage of distribution of 5hmC peaks, activation of INSIG1 by hypoxia in FADU cells, and TET1 knockdown abolished INSIG1 expression induced by hypoxia. [file 13059_2014_513_MOESM6_ESM.doc]
