# Supplementary material for: TET1 regulates hypoxia-induced epithelial-mesenchymal transition by acting as a co-activator
Source: Genome Biol. 2014 Dec 3;15(12):513. doi: 10.1186/s13059-014-0513-0 (PMC4253621; doi:10.1186/s13059-014-0513-0)

**Additional file 7: Figure S6. Localization of 5hmC peaks in the *FDPS* (a), *APOC1* (b), and *SQLE* (c) gene promoters by analysis of 5hmC sequencing.**


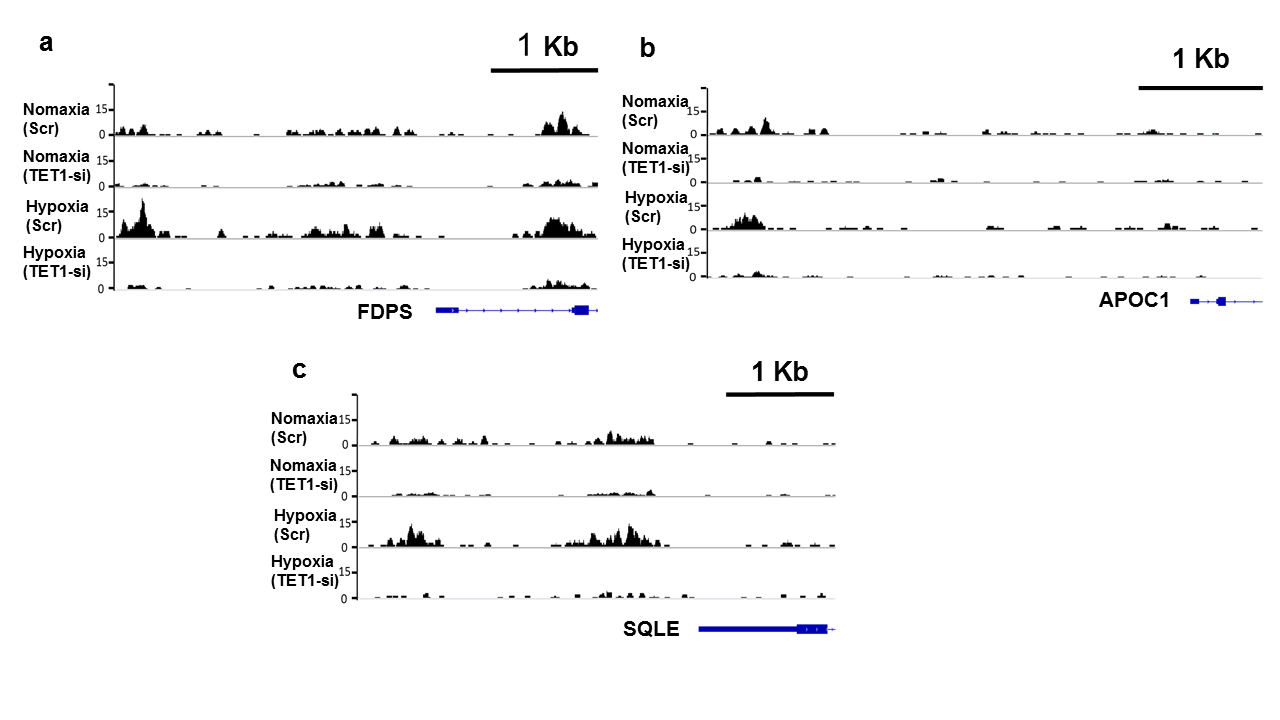

Supplement: Additional file 7: Figure S6. — Localization of 5hmC peaks in the FDPS (a), APOC1 (b), and SQLE (c) gene promoters by analysis of 5hmC sequencing. [file 13059_2014_513_MOESM7_ESM.doc]
