# Supplementary material for: TET1 regulates hypoxia-induced epithelial-mesenchymal transition by acting as a co-activator
Source: Genome Biol. 2014 Dec 3;15(12):513. doi: 10.1186/s13059-014-0513-0 (PMC4253621; doi:10.1186/s13059-014-0513-0)

**Additional file 8: Figure S7. 5hmC levels in the different promoter regions of *INSIG1* gene in H1299 cells or FADU cells under normoxia or hypoxia.** -920 to -847 and -1,455 to -1,364 represented the two regions in the promoter of *INSIG1* gene. The asterisk (*) indicates statistical significance (*P* <0.05) between experimental and control clones. The H299 scrambled control or H1299-TET1-si control clone under normoxia was chosen as the control condition in **(a)**. The FADU scrambled control or FADU-TET1-si control clone under normoxia was chosen as the control condition in **(b, c)**. Error bars indicate standard deviations (s.d.) of duplicate analysis by real-time PCR (a-c).


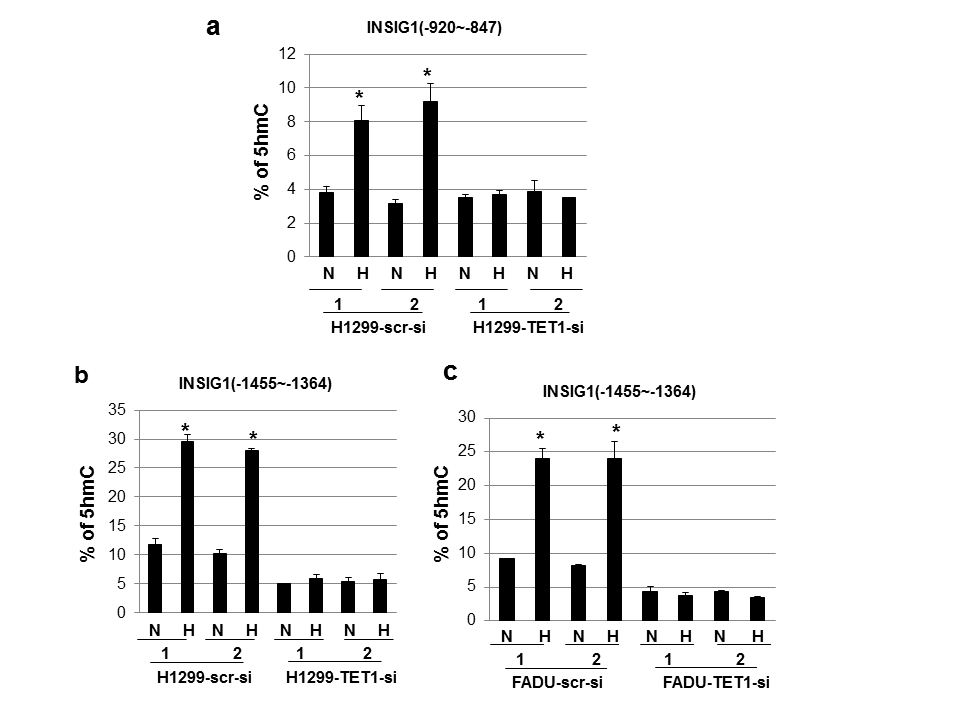

Supplement: Additional file 8: Figure S7. — 5hmC levels in the different promoter regions of INSIG1 gene in H1299 cells or FADU cells under normoxia or hypoxia. [file 13059_2014_513_MOESM8_ESM.doc]
