# Supplementary material for: TET1 regulates hypoxia-induced epithelial-mesenchymal transition by acting as a co-activator
Source: Genome Biol. 2014 Dec 3;15(12):513. doi: 10.1186/s13059-014-0513-0 (PMC4253621; doi:10.1186/s13059-014-0513-0)

**Additional file 10: Figure S9. Knockdown of INSIG1 mitigated hypoxia-induced epithelial-mesenchymal transition in H1299 cells, and the levels of Twist1 or Snail levels were not affected by INSIG1 knockdown in FADU cells. (a)** Knockdown of INSIG1 mitigated hypoxia-induced epithelial-mesenchymal transition in H1299 cells. **(b)** Knockdown of INSIG1 abolished the *in vitro* migration and invasion activity induced by hypoxia in H1299 cells. The asterisk (*) indicates statistical significance (*P* <0.05) between experimental and control clones. The control used was the first H1299 scrambled control clone under normoxia (either migration or invasion). Error bars indicate standard deviations (s.d.) of triplicate numbers counted from either migration or invasion. **(c)** Knockdown of INSIG1 did not affect the levels of Twist1 or Snail under hypoxia.


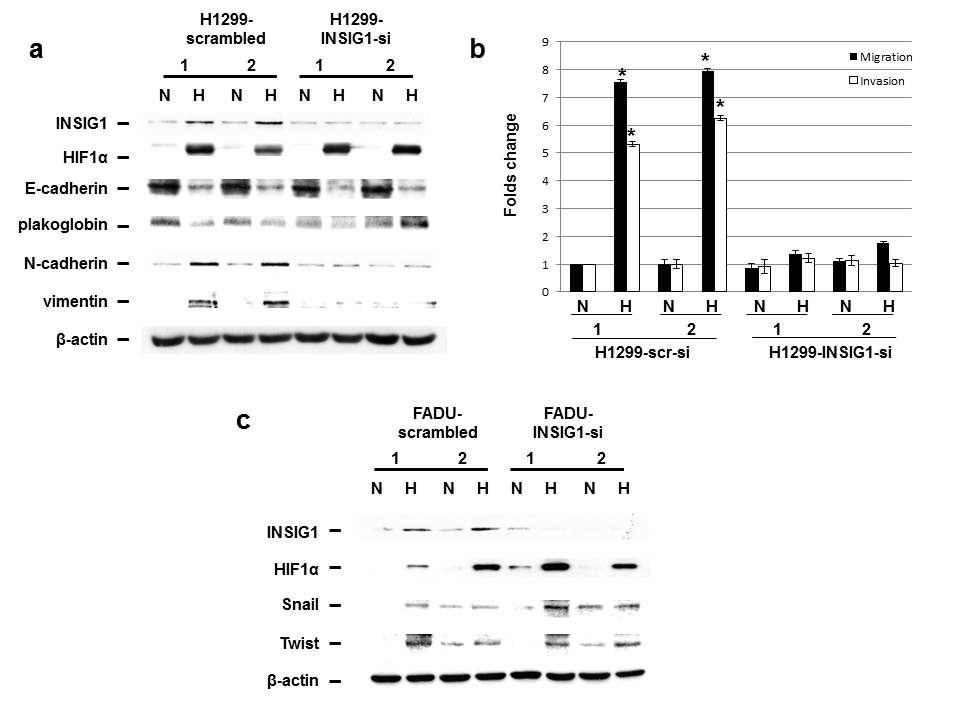

Supplement: Additional file 10: Figure S9. — Knockdown of INSIG1 mitigated hypoxia-induced epithelial-mesenchymal transition in H1299 cells, and the levels of Twist1 or Snail levels were not affected by INSIG1 knockdown in FADU cells. [file 13059_2014_513_MOESM10_ESM.doc]
