# Supplementary material for: TET1 regulates hypoxia-induced epithelial-mesenchymal transition by acting as a co-activator
Source: Genome Biol. 2014 Dec 3;15(12):513. doi: 10.1186/s13059-014-0513-0 (PMC4253621; doi:10.1186/s13059-014-0513-0)
Supplement: Additional file 11: Figure S10. — Real-time PCR analysis of the expression of various glycolytic enzymes (normoxia vs. hypoxia) under TET1 or INSIG1 knockdown. [file 13059_2014_513_MOESM11_ESM.doc]

**Additional file 11: Figure S10.** Real-time PCR analysis of the expression of various glycolytic enzymes (normoxia vs. hypoxia) under TET1 or INSIG1 knockdown in FADU cells. The asterisk (*) indicates statistical significance (*P* <0.05) between experimental (hypoxia) and control (normoxia) clones. The double asterisk (**) indicates statistical significance of GLUT3 gene (*P* <0.05) between experimental (hypoxia-INSIG1 knockdown) and control (hypoxia-scrambled) clones.

**
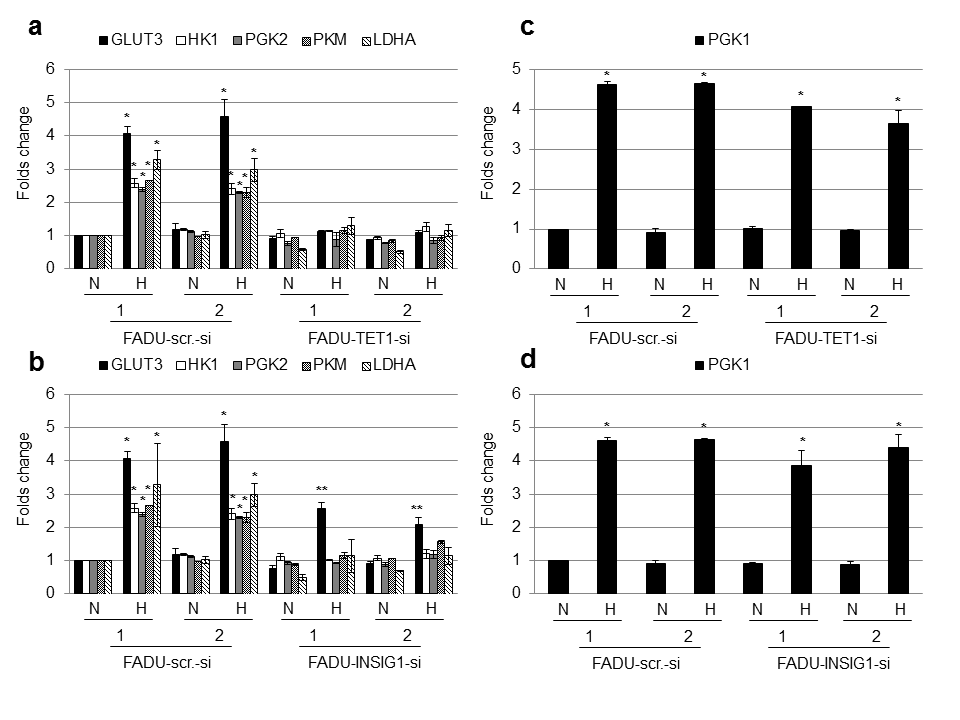
**
