# Supplementary material for: TET1 regulates hypoxia-induced epithelial-mesenchymal transition by acting as a co-activator
Source: Genome Biol. 2014 Dec 3;15(12):513. doi: 10.1186/s13059-014-0513-0 (PMC4253621; doi:10.1186/s13059-014-0513-0)

**Additional file 12: Figure S11. Synergistic activation of *WDR5* or *INSIG1* promoter by HIF-2α, TET1 (wild-type or point mutant), and CBP under normoxia or hypoxia. (a)** Synergistic activation of the *WDR5* promoter-driven reporter construct by HIF-2α, TET1 (wild-type or point mutant), and CBP. **(b)** Synergistic activation of the *INSIG1* promoter-driven reporter construct by HIF-2α and TET1 (wild-type or point mutant) under normoxia. **(c)** Synergistic activation of the *INSIG1* promoter-driven reporter construct by HIF-2α and TET1 (wild-type or point mutant) under normoxia or hypoxia. N: normoxia; H: hypoxia. The asterisk (*) indicates statistical significance (*P* <0.05) between experimental and control transfections. pXP2-WDR5 or pXP2-INSIG1 promoter-driven luciferase construct alone was used as the control transfection. Error bars indicate standard deviations (s.d.) of triplicate luciferase activity.


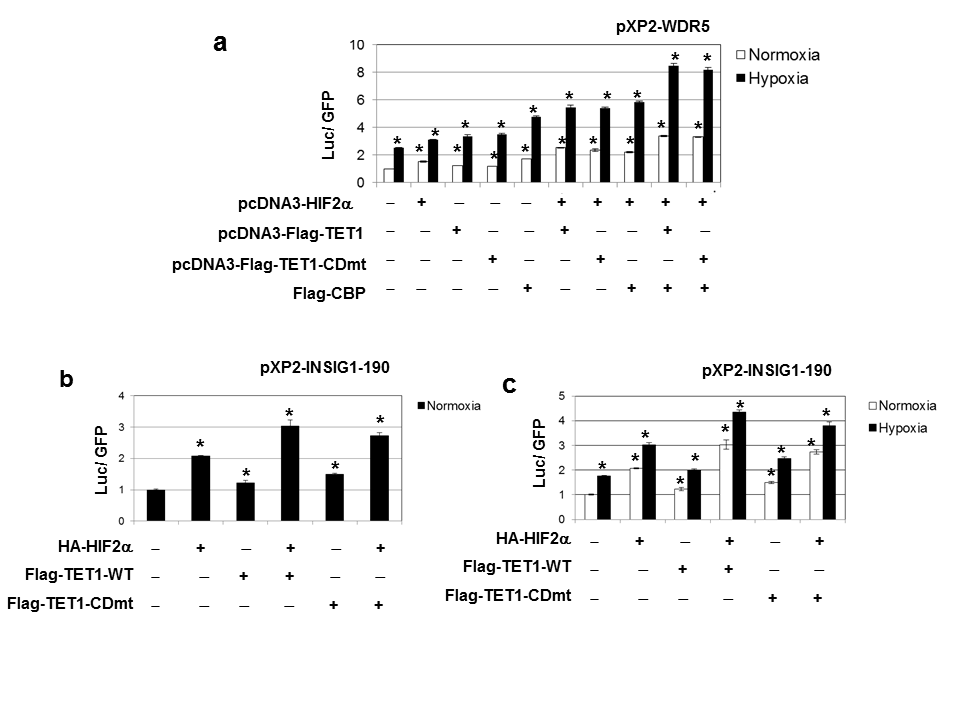

Supplement: Additional file 12: Figure S11. — Synergistic activation of WDR5 or INSIG1 promoter by HIF-2α, TET1 (wild-type or point mutant), and CBP under normoxia or hypoxia. [file 13059_2014_513_MOESM12_ESM.doc]
