# Supplementary material for: TET1 regulates hypoxia-induced epithelial-mesenchymal transition by acting as a co-activator
Source: Genome Biol. 2014 Dec 3;15(12):513. doi: 10.1186/s13059-014-0513-0 (PMC4253621; doi:10.1186/s13059-014-0513-0)

**Additional file 13: Figure S12. Co-immunoprecipitation experiments showed the interaction between TET1 and HIF-1α or HIF-2α.** **(a)** Co-immunoprecipitation assays showed that the anti-TET1 antibody pulled down HIF-1α. The TET1 point mutant (TET1-CDmt) also interacted with HIF-1α. **(b)** Co-immunoprecipitation assays showed that the anti-TET1 antibody pulled down HIF-2α. The TET1 point mutant (TET1-CDmt) also interacted with HIF-2α. WCE: whole cell extracts. **(c)** Co-immunoprecipitation assays showed that the anti-HA antibody against HA-HIF-2α pulled down TET1 and TET1-CDmt.


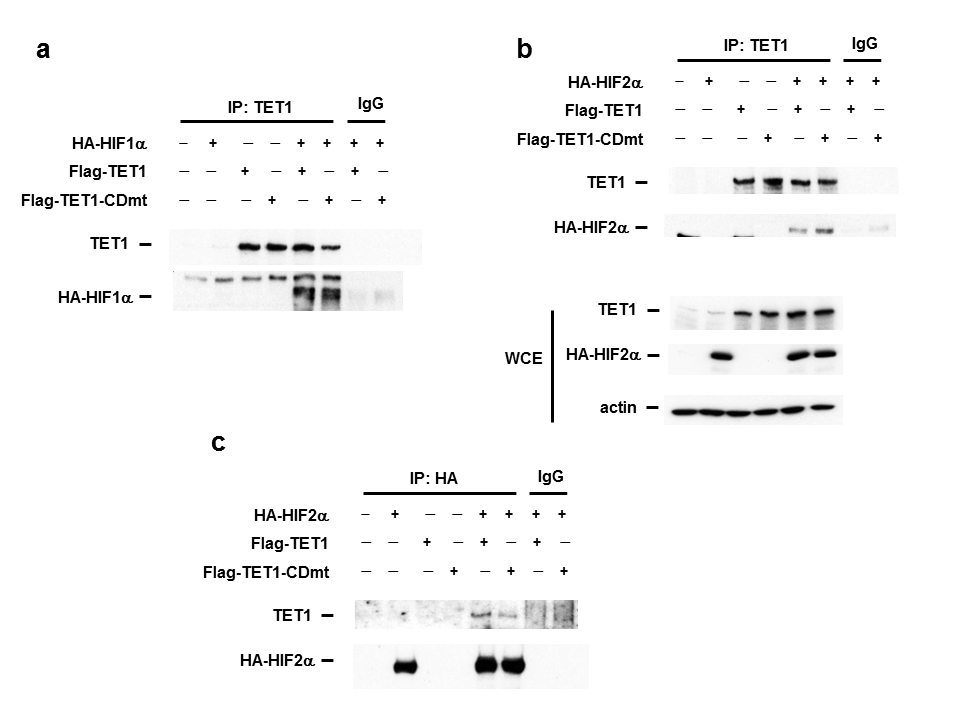

Supplement: Additional file 13: Figure S12. — Co-immunoprecipitation experiments showed the interaction between TET1 and HIF-1α or HIF-2α. [file 13059_2014_513_MOESM13_ESM.doc]
