# Supplementary material for: TET1 regulates hypoxia-induced epithelial-mesenchymal transition by acting as a co-activator
Source: Genome Biol. 2014 Dec 3;15(12):513. doi: 10.1186/s13059-014-0513-0 (PMC4253621; doi:10.1186/s13059-014-0513-0)

**Additional file 14: Figure S13. Mapping of the domain in HIF-1α that interacted with TET1.** **(a)** The expression of TET1, HIF-1α, and two HIF-1α truncation mutants used in co-immunoprecipitation experiments. WCE: whole cell extracts. **(b)** Co-immunoprecipitation assays showed that the full length HIF-1α and HIF-1α-1-400 truncation mutant interacted with TET1.


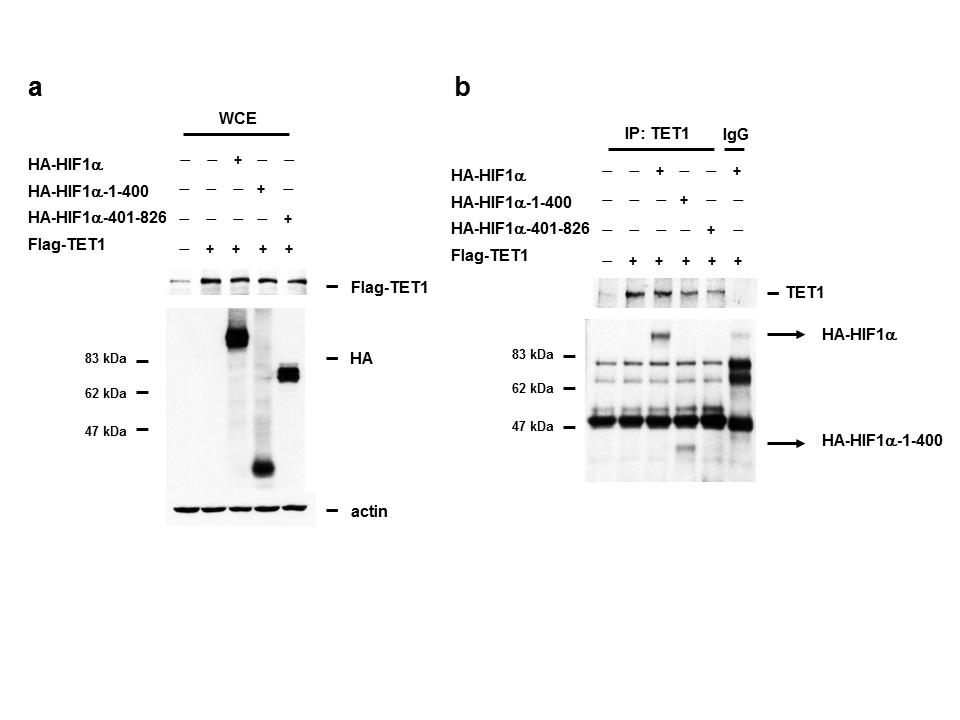

Supplement: Additional file 14: Figure S13. — Mapping of the domain in HIF-1α that interacted with TET1. [file 13059_2014_513_MOESM14_ESM.doc]
