# Supplementary material for: TET1 regulates hypoxia-induced epithelial-mesenchymal transition by acting as a co-activator
Source: Genome Biol. 2014 Dec 3;15(12):513. doi: 10.1186/s13059-014-0513-0 (PMC4253621; doi:10.1186/s13059-014-0513-0)

**Additional file 15: Figure S14. Further mapping of the domain in HIF-1α that interacted with TET1 and dissection of the TET1 transactivation domain. (a)** The HIF-1α-81-400 truncation mutant interacted with TET1. Upper panel represented the expression levels of various proteins. Lower panel represented the co-immunoprecipitation assays. WCE: whole cell extracts. **(b)** The HIF-1α-175-305 truncation mutant interacted with TET1. Upper panel represented the expression levels of various proteins. Lower panel represented the co-immunoprecipitation assays. WCE: whole cell extracts.**(c)** Disruption of the TET1 a.a. 451 to 674 domain abolished the transactivation activity of TET1 using yeast one hybrid assays.


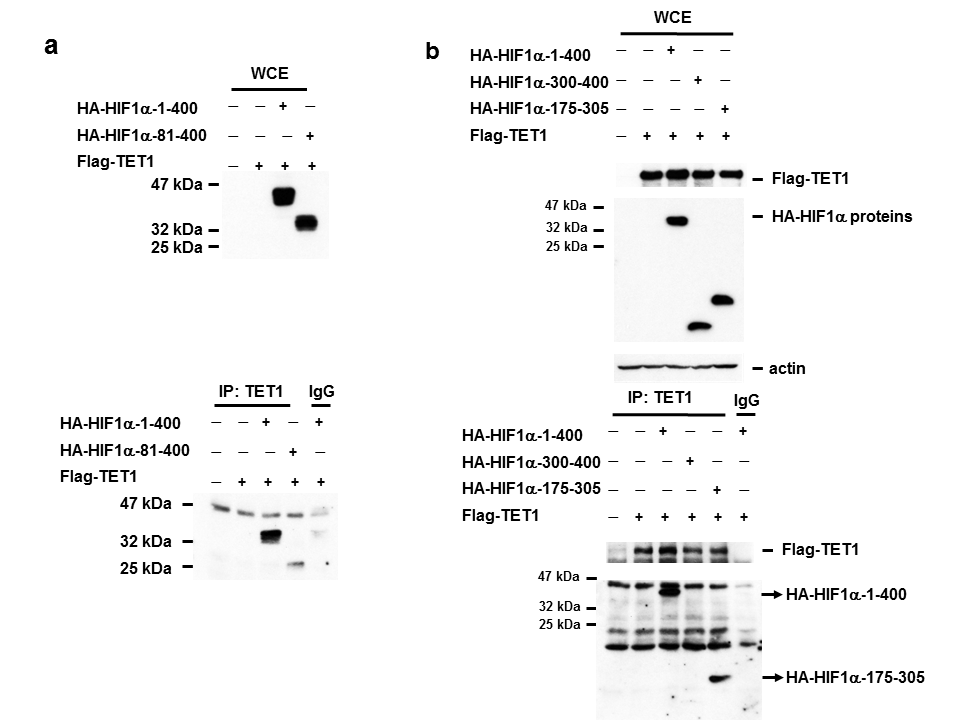


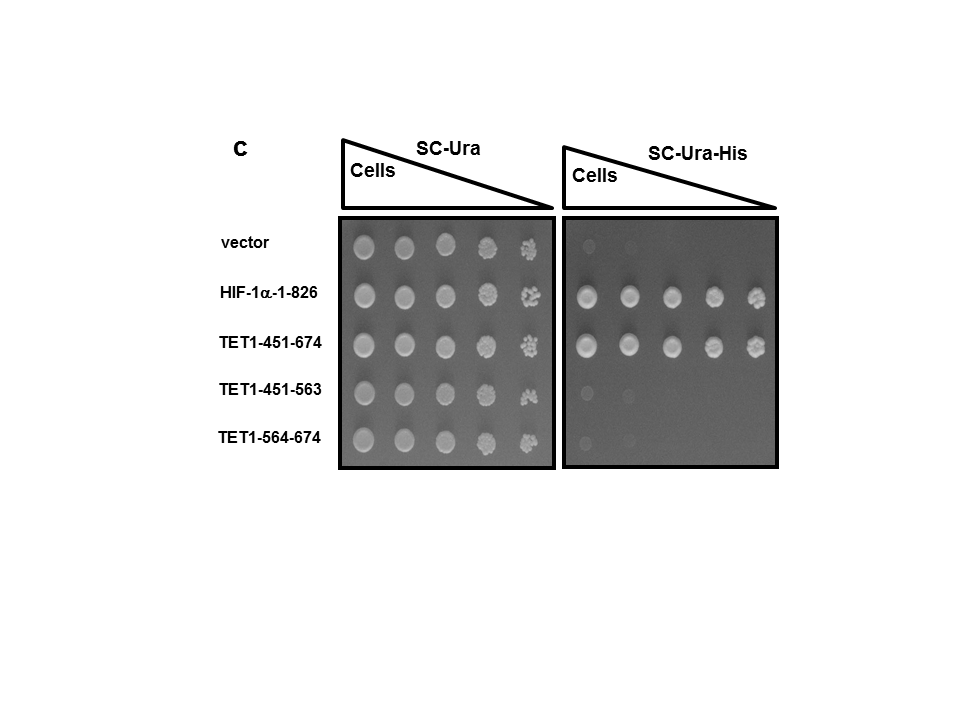

Supplement: Additional file 15: Figure S14. — Further mapping of the domain in HIF-1α that interacted with TET1. [file 13059_2014_513_MOESM15_ESM.doc]
