# Supplementary material for: TET1 regulates hypoxia-induced epithelial-mesenchymal transition by acting as a co-activator
Source: Genome Biol. 2014 Dec 3;15(12):513. doi: 10.1186/s13059-014-0513-0 (PMC4253621; doi:10.1186/s13059-014-0513-0)
Supplement: Additional file 16: Figure S15. — Various co-immunoprecipitation experiments to check the interaction between different proteins. [file 13059_2014_513_MOESM16_ESM.doc]

**Additional file 16: Figure S15.** Various co-immunoprecipitation experiments to check the interaction between different proteins.

**
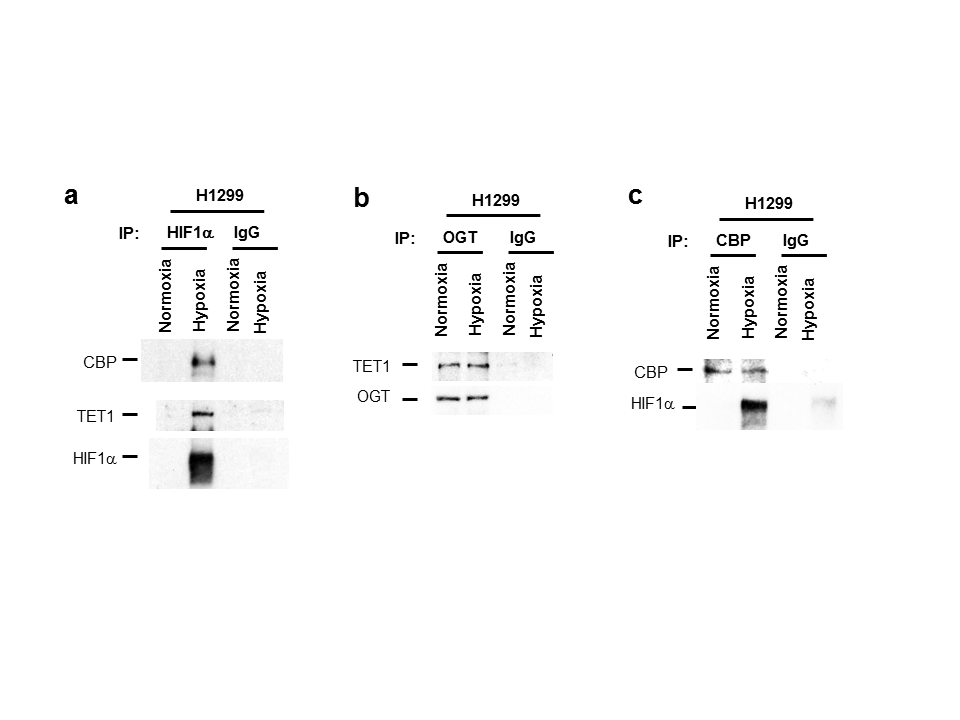
**
