# Supplementary material for: TET1 regulates hypoxia-induced epithelial-mesenchymal transition by acting as a co-activator
Source: Genome Biol. 2014 Dec 3;15(12):513. doi: 10.1186/s13059-014-0513-0 (PMC4253621; doi:10.1186/s13059-014-0513-0)
Supplement: Additional file 17: Table S2. — List of proteins tested by antibodies and characteristics of the corresponding antibodies. [file 13059_2014_513_MOESM17_ESM.doc]

**Additional file 17: Table S2. List of proteins tested by antibodies and characteristics of the corresponding antibodies**

| **Protein** | **Assay** | **Antibody** | **Origin** | **Dilution** | **Incubation period** |
| --- | --- | --- | --- | --- | --- |
| 5-methylcytidine | MeDIP | mmab | BI-MECY-0100, Eurogentec | 1:50 | 4°C, overnight |
| β-actin | WB | mmab | A5441, Sigma-Aldrich Co. | 1:10,000 | 4°C, overnight |
| CBP | WB | mmab | sc-7300, Santa Cruz | 1:200 | 4°C, overnight |
| CBP | IP | mmab | sc-7300, Santa Cruz | 25 μL | 4°C, 4 h |
| E-cadherin | WB | rpab | NG1848919, Millipore | 1:1,000 | 4°C, overnight |
| Flag | WB | mmab | F3165, Sigma Aldrich Co. | 1:1,000 | 4°C, overnight |
| γ-catenin | WB | mmab | Cat.610253, BD | 1:500 | 4°C, overnight |
| HA | WB | mmab | # 2367S, Cell signaling | 1:500 | 4°C, overnight |
| HA | IP, ChIP | mmab | 05-904, Millipore | 5 μL | 4°C, 4 h |
| HIF1α | WB | rpab | GTX1273090, GeneTex | 1:1,000 | 4°C, overnight |
| HIF1α | WB | mmab | Cat.610959, BD | 1:1,000 | 4°C, overnight |
| HIF2α | WB | rpab | GTX128193, GeneTex | 1:1,000 | 4°C, overnight |
| INSIG1 | WB | rpab | ab70784, abcam | 1:200 | 4°C, overnight |
| N-cadherin | WB | mmab | Cat.610921, BD | 1:250 | 4°C, overnight |
| OGT | WB | rpab | sc-32921, Santa cruz | 1:1,000 | 4°C, overnight |
| OGT | IP | rpab | sc-32921, Santa cruz | 25 μL | 4°C, 4 h |
| TET1 | WB | mmab | GTX627420, GeneTex | 1:500 | 4°C, overnight |
| TET1 | IP | mmab | GTX627420, GeneTex | 5 μL | 4°C, 4 h |
| Vimentin | WB | mmab | 090M4817, Sigma | 1:500 | 4°C, overnight |
