# Supplementary material for: TET1 regulates hypoxia-induced epithelial-mesenchymal transition by acting as a co-activator
Source: Genome Biol. 2014 Dec 3;15(12):513. doi: 10.1186/s13059-014-0513-0 (PMC4253621; doi:10.1186/s13059-014-0513-0)
Supplement: Additional file 18: Table S3. — Sequence of the oligonucleotides for real-time PCR. [file 13059_2014_513_MOESM18_ESM.doc]

**Additional file 18: Table S3. Sequence of the oligonucleotides for real-time PCR**

| **Target** | **Sequence (5' → 3')** |
| --- | --- |
| TET1 | F: TCTGTTGTTGTGCCTCTGGA |
|  | R: CTGGTTTGTTGTCAAAATCTGCCTT |
| 18 S | F: GGCGGCGTTATTCCCATGA |
|  | R: GAGGTTTCCCGTGTTGAG |
| VEGF | F: GGCGGCGTTATTCCCATGA |
|  | R: GTGATGTTGGACTCCTCAGT |
| GLUT3 | F:GACCCAGAGATGCTGTAATGGT R:TGGCAAATATCAGAGCTGGGG |
| HK1 | F:TGGCCTATTACTTCACGGAGC  R:GTTTCATCGGAGAGCCGCAT |
| PGK2 | F:TCCATGGCCCTTAAGTCAGC R:TCCATGGCCCTTAAGTCAGC |
| PKM | F:GAGGCCTCCTTCAAGTGCTG R:GACGAGCTGTCTGGGGATTC |
| LDHA | F:CATGGCCTGTGCCATCAGTA R:AGATATCCACTTTGCCAGAGAC |
| PGK1 | F:CCACTGTGGCTTCTGGCATA  R:ATGAGAGCTTTGGTTCCCCG |
| HIF2α | F: CCTGCGAACACACAAGCTCCTCTCC |
|  | R: CCACGGCAATGAAACCCTCCAAGG |
| INSIG1 | F: GACATTTGATCGTTCCAGAAGTGG |
|  | R: CACCATTATACACGAGAAACTGCG |
