# Supplementary material for: TET1 regulates hypoxia-induced epithelial-mesenchymal transition by acting as a co-activator
Source: Genome Biol. 2014 Dec 3;15(12):513. doi: 10.1186/s13059-014-0513-0 (PMC4253621; doi:10.1186/s13059-014-0513-0)
Supplement: Additional file 19: Table S4. — Sequence of the oligonucleotides for construct-making. [file 13059_2014_513_MOESM19_ESM.doc]

**Additional file 19: Table S4. Sequence of the oligonucleotides for construct-making**

| **Constructions )** | **Sequence (5' → 3')** | **Restriction site** |
| --- | --- | --- |
| pXP2-INSIG1-190 | F: CCCAAGCTTGGCCAGTCCCGCTCGGGC | HindIII |
| (+1 to -190) | R: GAAGATCTGCCTGAGCTTGCCCCGCCCC | BglII |
| pXP2-TET1-158 | F: AAGCTTTGGAGAACGAGGGGGAG | HindIII |
| (+17 to -158) | R: AGGTCAGATCTCCGGAGCAGCAGTGTCT | BglII |
| pXP2-TET1-91 | F: CAGTTAAGCTTAATCCAGCTCGCGT | HindIII |
| (+17 to -91) | R: AGGTCAGATCTCCGGAGCAGCAGTGTCT | BglII |
| pGBDU-C1-TET1-675-1579 | F1: AATTCAGTAGATGTGGTCATGGGGA | EcoR1 |
|  | F2: CAGTAGATGTGGTCATGGGGA | EcoR1 |
|  | R1: GATCCTCAACAGCCAAAAGAGAATGAAG | BamH1 |
|  | R2: CTCAACAGCCAAAAGAGAATGAAG | BamH1 |
| pGBDU-C1-TET1-1580-2136 | F: CTTTTGAATTCATGTGGAGTATGTACTTTAATGGC | EcoR1 |
|  | R: CGGGATCCTCAGACCCAATGGTTATAG | BamH1 |
| pGBDU-C1-TET1-1-225 | F1: AATTCATGTCTCGATCCCGCCAT | EcoR1 |
|  | F2: CATGTCTCGATCCCGCCAT | EcoR1 |
|  | R1: GATCCTCATTCACCACAGCGTGTCCCT | BamH1 |
|  | R2: CTCATTCACCACAGCGTGTCCCT | BamH1 |
| pGBDU-C1-TET1-226-450 | F1: AATTCGGACTATTCTCTGAAGAG | EcoR1 |
|  | F2: CGGACTATTCTCTGAAGAG | EcoR1 |
|  | R1: GATCCTCAAGGCCATTTGGAAGGAGCA | BamH1 |
|  | R2: CTCAAGGCCATTTGGAAGGAGCA | BamH1 |
| pGBDU-C1-TET1-451-674 | F1: AATTCGAGCCCCAAAGCACTGTC | EcoR1 |
|  | F2: CGAGCCCCAAAGCACTGTC | EcoR1 |
|  | R1: GATCCTCAGTAGTCCATGGATTCTGA | BamH1 |
|  | R2: CTCAGTAGTCCATGGATTCTGA | BamH1 |
| pGBDU-C1-TET1-451-563 | F: CTTCCGAATTCATGGAGCCCCAAAGCACTGTCTCA | EcoRI |
|  | R: ATTGGGGATCCCTAAGTCACCACTGTGGTGTTGAC | BamHI |
| pGBDU-C1-TET1-564-674 | F: ACAGTGGAATTCATGCCAGTGCCAATGGTCAGT | EcoRI |
|  | R: TGACCGGATCCCTAGTAGTCCATGGATTCTGACT | BamHI |
| pGBDU-C1-HIF1α-1-826 | F: GACCGAGGATCCATGGAGGGCGCCGGCGGC | BamHI |
|  | R: AAATTAATCGATAGCTCAGTTAACTTGATCCAAAGC | ClaI |
