# Supplementary material for: TET1 regulates hypoxia-induced epithelial-mesenchymal transition by acting as a co-activator
Source: Genome Biol. 2014 Dec 3;15(12):513. doi: 10.1186/s13059-014-0513-0 (PMC4253621; doi:10.1186/s13059-014-0513-0)
Supplement: Additional file 20: Table S5. — Sequence of the oligonucleotides for ChIP analysis. [file 13059_2014_513_MOESM20_ESM.doc]

**Additional file 20: Table S5. Sequence of the oligonucleotides for ChIP analysis**

| **Target** | **Sequence (5' → 3')** |
| --- | --- |
| TET1 (- 114 to - 9) | F: AGGGATTCCAGCTCCAGTTT |
|  | R: CAGACCTCAGGGAGTGAAGC |
| WDR5 (- 608 to - 884) | F: TGAAGCAGGCGGCGAGAATG |
|  | R: TTCCCGCCTCTGCATGCACAGA |
