# Supplementary material for: TET1 regulates hypoxia-induced epithelial-mesenchymal transition by acting as a co-activator
Source: Genome Biol. 2014 Dec 3;15(12):513. doi: 10.1186/s13059-014-0513-0 (PMC4253621; doi:10.1186/s13059-014-0513-0)
Supplement: Additional file 21: Table S6. — Sequence of the lentiviral siRNA vectors. [file 13059_2014_513_MOESM21_ESM.doc]

**Additional file 21: Table S6. Sequence of the lentiviral siRNA vectors**

| **Target gene** | **Sequence (5' → 3')** |
| --- | --- |
| TET1 | CCTTGATAGAATCACTCAGTT |
| HIF1α | GTGATGAAAGAATTACCGAAT |
| HIF2α | CAGTACCCAGACGGATTTCAA |
| INSIG1 | CGGCAATGATTTCTTTCAGTT |
