# Supplementary material for: TET1 regulates hypoxia-induced epithelial-mesenchymal transition by acting as a co-activator
Source: Genome Biol. 2014 Dec 3;15(12):513. doi: 10.1186/s13059-014-0513-0 (PMC4253621; doi:10.1186/s13059-014-0513-0)
Supplement: Additional file 22: Table S7. — Sequence of the oligonucleotides for 5hmc assay and MeDIP. [file 13059_2014_513_MOESM22_ESM.doc]

**Additional file 22: Table S7. Sequence of the oligonucleotides for** 5hmc assay and MeDIP

| **Target** | **Sequence (5' → 3')** |
| --- | --- |
| INSIG1(-1455 to -1364) | F: CTGGCACTTGCTGGTGTTTC |
|  | R: GCTAACCTCATGGAGAGGCG |
| INSIG1(-920 to -847) | F: GCGTGTCATCCTCAGGAAAT |
|  | R: GAGCTGAGACTGCCCTGAGT |
